# Active Learning of Atomic Size Gas/Solid Potential Energy Surfaces via Physics Aware Models

**DOI:** 10.1021/acs.jcim.5c01193

**Published:** 2025-08-12

**Authors:** Nikolaos Patsalidis, Mohsen Doust Mohammadi, Somnath Bhowmick, George Biskos, Vagelis Harmandaris

**Affiliations:** † Computation-based Science and Technology Research Center, 338376 The Cyprus Institute, Aglantzia 2121, Cyprus; ‡ Climate & Atmosphere Research Centre, 338376 The Cyprus Institute, Aglantzia 2121, Cyprus; § Department of Mathematics and Applied Mathematics, 37777 University of Crete, Heraklion GR-71110, Greece; ∥ Institute of Applied and Computational Mathematics, Foundation for Research and Technology—Hellas, Heraklion GR-71110 Crete, Greece

## Abstract

We propose an active learning (AL) framework to develop classical force fields (FFs) that accurately model the potential energy surfaces (PES) of gas/solid atomic-scale complexes. A central challenge is integrating AL with flexible, computationally efficient physics-aware potentials to achieve quantum-level accuracy for complex interfacial systems. Our approach trains physics-aware potentials, with incorporated flexibility and smoothness, on actively sampled density functional theory (DFT) data to describe interactions between undercoordinated atomic silver (Ag) clusters and gaseous pollutants (CO_2_, CO, SO_2_), relevant for environmental applications like sensing. The AL process follows three stages: (1) FFs are trained using adaptable physics aware potentials of semiempirical descriptors, optimized via a Pareto analysis scheme; (2) new candidate structures are generated through the use of the refined FFs in Metropolis Hastings Monte Carlo (MHMC) or stochastic molecular dynamics (sMD) simulations; (3) a subset of candidates is selected for DFT computations based on an outlier score (OS), which utilizes the existing data descriptor distributions, ensuring diverse PES exploration. This framework produces FFs capable of capturing cohesive, physisorption, and chemisorption interactions with admirable accuracy, close to ab initio methods, while retaining the efficiency of semiempirical potentials. To demonstrate, produced FFs are utilized in molecular dynamics (MD) simulations of single Ag clusters embedded in bulk gas phases, examining condensation characteristics. Our methodology is highly versatile, easily accommodating various choices of descriptors, model basis sets, and sampling techniques.

## Graphical abstract

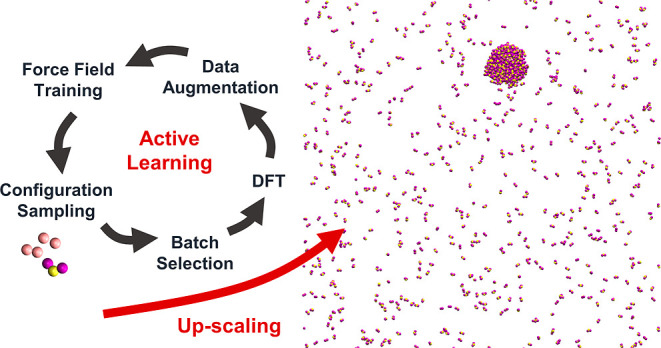

## 1 Introduction

The holy grail in the development of atomistic models, necessary for performing atomistic molecular dynamics (MD) or Monte Carlo (MC) simulations, is to obtain classical force fields (FFs) of high accuracy and computational efficiency. A promising route to address this challenge is based on active learning (AL) approaches. These involve strategic selection of quantum chemical data, often obtained through computationally expensive density functional theory (DFT) calculations, and the active training and refining of machine-learned FFs on diverse data, generated via efficient exploration schemes of the underlying potential energy surface (PES).^1–8^ Such machine-learned FFs, although highly accurate, present high computational costs compared to semiempirical potentials that rely on physically justified potential energy terms when applied to long MD simulations.^9–13^ At the same time, for MD simulations to have a profound impact on material properties prediction and design, long simulations, nowadays of the order of microseconds (μs), are required. Therefore, semiempirical potentials are practically still used in the majority of simulation studies for complex molecular systems. An important question is whether AL approaches can be effectively combined with low-cost, physics-informed models.

Any atomistic model that describes the PES of an underlying molecular system via predefined descriptors of the atomic environment should have certain properties. These include invariances to Euclidean transformations (translation, rotation, reflection) and permutation of chemically equivalent atoms, FF smoothness (implies continuity), and (the most challenging) faithfulness; that is, each configuration should be described by a unique descriptor.^14–16^ In practice, besides faithfulness, the above properties are inherently satisfied by the choice of the descriptor and the potential form, i.e., the functional basis set used to describe the underlying PES. For example, the descriptors in popular semiempirical FFs used in soft matter, like OPLS,^10^ and COMPASS,^12^ are the bond lengths, the bond angles, the dihedral angles, and pairwise distances between nonbonded atoms. Models such as the embedded atom model (EAM) and modified EAM,^11,13^ applied in solid-state of matter, such as metals, include many-body interactions via a scalar density descriptor, which counts the effective number of neighbors of each atom by expanding on pairwise distances and, hence, preserving pairwise-like efficiency.

FFs developed by machine learning (ML) methods are practically extended functional basis sets describing n-body interactions, which are of quantum origin, at the classical level.^14–26^ Functional many-body representations, such as the smooth overlap of atomic positions (SOAP), are commonly used in kernel-based ML methods to predict by interpolation energies and forces, using the similarity of the new descriptor vector with the reference data.^15,23,27–29^ In a similar way, neural network (NN) models are used to represent many-body interactions, usually using complex nonlinear mappings of atomic descriptors to accurately compute the interaction potential.^5,14,17,22,24,25^ Such ML models demonstrate high accuracy (1–10 meV/atom), but their computational cost scales linearly with the size of the reference data or of the NN. They are also prone to overfitting and may predict unrealistic energy values when extrapolating beyond their training domain.^1,17,21,27,29–31^ Finally, significant improvement in the extrapolation ability of the model could be achieved by considering physics-aware 2- and 3-body descriptors, like pairwise distances, in combination with extended ML basis sets.^21^

Extrapolation and overfitting issues show that adequately sampling the PES is crucial. To address this, AL algorithms are often used to ensure diversity in training data.^1,2,4–7,31–33^ For instance, Vandermause et al.^31^ used the inherent uncertainty in a Gaussian process regression model to decide whether to accept its prediction or perform a DFT calculation during temperature-annealing MD simulations of crystalline materials. Kulichenko et al.^4^ used metadynamics to sample different local minima of the PES and estimated model uncertainty via the disagreement among multiple NN models capturing proton transfer in glycerine. Wilson et al.^32^ developed a batch AL scheme that enhances the training set with various structures selected from a pool of data generated by ab initio MD simulations of germanium selenide (GeSe). In each AL iteration they selected the most dissimilar structures with existing data, based on the Euclidean distance of the feature vectors. Moreover, their model was based on 2- and 3-body terms resembling semiempirical forms. In another recent work, Bernstein et al.^6^ developed a batched AL scheme that samples new data based on structural dissimilarity, using energy minimization trajectories to explore different PES local minima and, hence, stable structures of crystalline materials.

The studies referred above are very promising, but they consider either crystalline solid-state phases, small isolated molecules, or water clusters. In contrast, to the best of our knowledge, complex interfaces and/or undercoordinated hybrid materials have not yet been addressed. Examples include systems associated with important technological applications that rely on molecules adsorbed on surfaces^34^ and gas/metal hybrids,^35^ relevant to emerging environmental challenges such as pollutant detection, clean energy production and decarbonization. For example, atomic silver nanoclusters are very promising for gas sensing applications due to the different gas adsorption characteristics, which could be either of physisorption, or chemisorption character and of different interaction strength.^35^ Moreover, they can be readily produced by innovative gas-phase synthesis methods, such as atmospheric-pressure spark ablation.^36^ These systems are extremely challenging in terms of modeling, as they exhibit an extremely rough PES and are characterized by a variety of complex quantum chemical interactions within scales of a few Å, Moreover, adsorption, desorption or nucleation (around solid nanoclusters) phenomena occur on a wide range of time scales, from a few fs that corresponds to adsorption of single molecules to atomic clusters, up to several μs, relevant to cluster nucleation.^37,38^

To address the above challenges, the development of accurate and preeminently efficient atomistic FFs is essential. Moreover, the FF basis set should be tunable to different characteristic interactions and, ideally, transferable over a range of systems with different cluster sizes and temperatures. Additionally, the potential form should be generic and systematically improvable through a suitable AL framework that efficiently explores the PES.

Here we employ a batch AL strategy that actively trains generalized forms of semiempirical potentials by exploring and sampling the PES at the DFT level. We apply it to a variety of complex interfacial systems, i.e., isolated silver (Ag) atomic clusters (of 7–16 atoms), and on CO_2_/Ag, CO/Ag, SO_2_/Ag undercoordinated atom sized hybrids. The FF basis set follows physically aware descriptors, e.g., bond distances and bond angles, pairwise distances between atoms and embedding densities that describe the local environment. Their dependency to the potential energy is direct, similar to the physically aware semiempirical potential forms like Morse. Moreover, our model also incorporates flexibility by considering potential curves of arbitrary shape, parametrized via, respectively, the flexible Bezier polynomials^39^ (see Sections 2.1 and 2.2). Such parametrization allows for a smooth (continuously differentiable) and controllable potential that, with only a few extra training parameters, can capture complex underlying physicochemical interactions. Using Bezier polynomials, we include flexible pairwise corrections and flexible many-body potential terms. Part of the model captures the essential physics, while another part introduces the necessary flexibility to fit complex quantum deviations. In addition, this model maintains pairwise efficiency and, as we will show, it is transferable with a small trade-off in accuracy across different atomic cluster sizes. Even more importantly, the derived family of models is capable of describing very rough PES of the atom-sized gas/metal hybrids with quantum accuracy, via relatively simple physics-aware cost-efficient basis functions, without the need to consider more complex descriptors commonly used in ML FFs.^17^ Finally, we demonstrate the scalability and applicability of this approach by performing MD simulations of single Ag clusters embedded in the bulk gas phase.

## 2 Methods

The overall AL methodology is summarized in Figure 1. First, an initial data set is used for training. Due to numerical noise of the DFT data (data uncertainty) and perhaps inadequacies of the model (model or epistemic uncertainty), minimizing the differences between improving the fit on the force labels may worsen the fit in energy labels and vice versa. Our training method minimizes the energy cost at descending values of forces cost, finding Pareto optimal solutions. We keep the solution that exhibits the best trade-off between the energy cost and the force cost (see Section 2.3). In the next step, the trained or refined FF is used to generate a large pool of new candidate gas/cluster hybrid structures (here 40,000) via either Metropolis Hastings Monte Carlo (MHMC) or stochastic molecular dynamics (sMD) simulations (see Section 2.4). Since they are generated using the model, these candidates are unlabeled (no ground truth values of energies and forces). From these, a small subset of structures (here a batch of 200) is selected with a probability proportional to each candidate’s outlier score (OS). The latter is quantified based on the overlap of each new descriptor to the corresponding descriptor distribution of the existing data. This algorithm promotes selection of out-of-distribution or rarely occurring structures (see Section 2.5), and hence, the structures that have not yet been “seen” during training. In the final step, the selected data undergo DFT labeling and are used for performance evaluation and augmentation of the data set for the next AL iterations. This is an effective way to actively sample and explore new regions of the PES, augmenting the data set with diverse structures. The labeled data set at each AL iteration is referred as existing (e).

**Figure 1:**
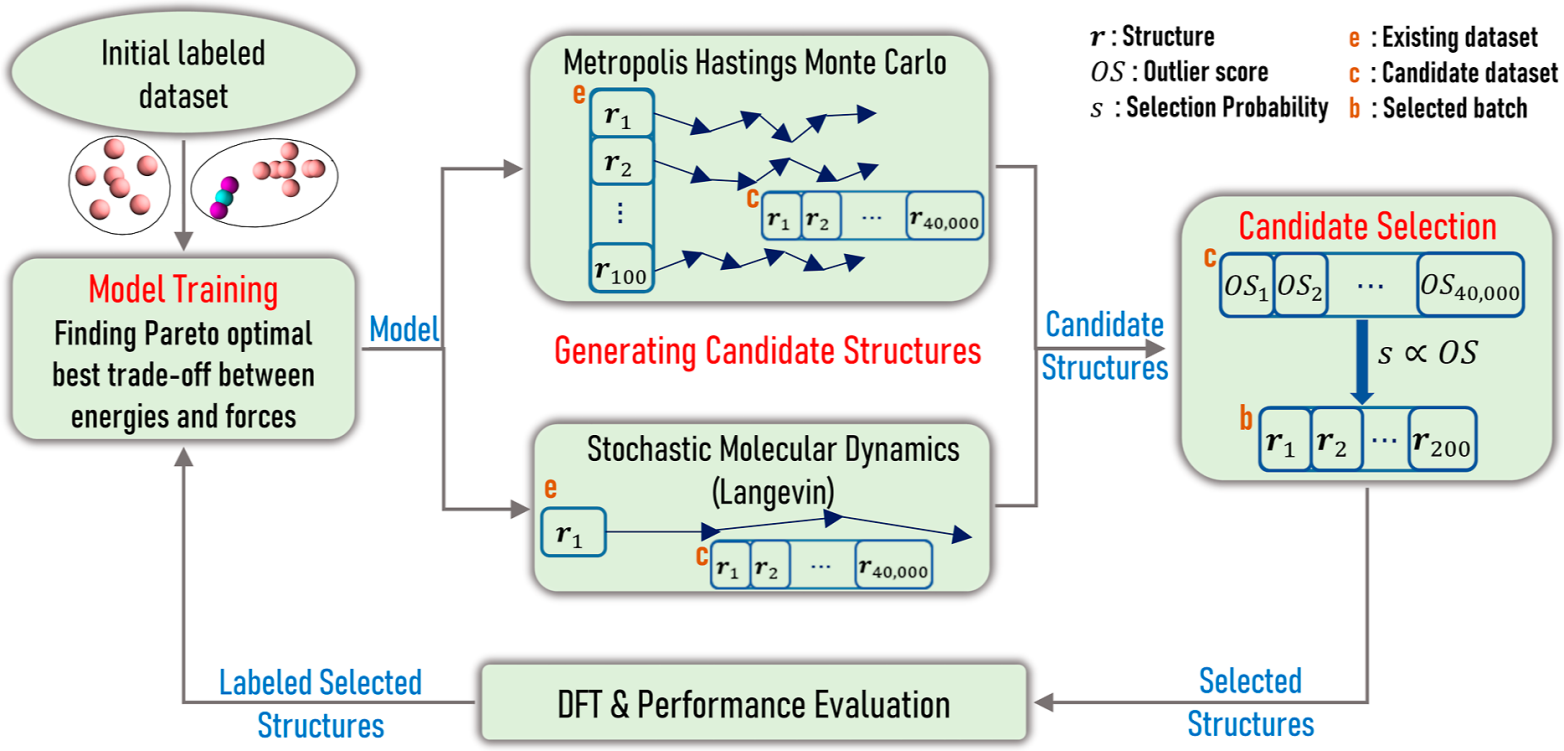
Schematic of the active learning (AL) methodology. Beginning from an initial labeled data set, with as many as 1 structure per system, we train the model by finding Pareto optimal solutions and choosing the one that shows the best trade-off between the energy cost and forces cost. In the second step, using either Metropolis Hastings Monte Carlo (MHMC) or stochastic molecular dynamics (sMD) a large pool of candidate structures (here, 40,000) is generated. MHMC is performed on 100 parallel paths, starting from respectively 100 initial configurations, chosen randomly from the existing labeled data set. A single longer trajectory is acquired using sMD, starting respectively from 1 random configuration from the existing data set. In the next step, for each candidate, an outlier score (OS) is computed and a subset (batch) of these structures is selected (here 200), via a probability proportional to OS. DFT is performed on the selected structures to label their energies and forces. The newly labeled batch is used for performance evaluation and augmenting the existing data set in the next AL iterations.

The proposed AL-based methodology does not explicitly depend on the model itself, nor on the type of the descriptors. Any model, functional basis set, and any set of descriptors could be used that respect the permutational, translation, and rotational invariance, such as NNs that utilize symmetry functions as their input descriptors or kernel methods.^14,15,25^ Finally, our algorithm effectively generates the training data, since it can be initiated by just one point per chemical system, as we demonstrate in Section 3. In this context, exploiting and labeling out-of-distribution candidates becomes even more important, since ideally we would like to perform as fewer DFT calculations as possible.

The subsections below describe in detail (a) the classical representation of the complex gas/cluster hybrid PES (classical model) and (b) its parametrization, each step of the AL methodology, including (c) the training procedure, (d) the candidate sampling methods, (e) the OS quantification method, and (f) the data labeling methods, DFT settings and performance metric. Finally, (g) we provide a small description of the AL initialization and the code.

### 2.1 Classical Description of Gas/Cluster PES

The initial part of any classical model concerns the choice of the appropriate descriptors that describe the underlying many-body, of quantum origin, PES. Here a physics aware parametrization of the complex PES of the gas/atomic cluster hybrid is proposed as the sum of bonded (*U*_B_(**r**)), pairwise nonbonded (*U*_PW_(**r**)) and embedding (*U*_E_(**r**)) contributions, using the following functional basis set

1 $$U_{class}\left(r\right)=U_{B}\left(r\right)+U_{PW}\left(r\right)+U_{E}\left(r\right)$$

The bonded terms in the above expression include interactions describing chemical bonds, bond angles, and dihedral angles as

2 $$U_{B}\left(r\right)=\sum_{b}f_{b}\left(r_{b}\right)\sum_{a}f_{a}\left(\theta_{a}\right)$$

where *r*_b_ is the length of the bond indexed by b, θ_a_ the bond angle indexed by a, and *f*_b_, *f*_a_, are functions used to describe interactions between chemical bonds and angles, respectively. The pairwise terms are of the general form

$$U_{PW}\left(r\right)=\underset{i<j}{\sum }f_{pw}\left(r_{ij}\right)$$ 3

where *r*_*ij*_ is the distance between nonbonded atoms *i* and *j* and *f*_pw_ the functions that describe the pairwise interactions. Last, the embedding contributions that typically describe the many-body terms that correspond to the local chemical environment

4 $$U_{E}\left(r\right)=\sum_{i,\beta \in B_{i}}f_{e}\left(\rho_{\alpha \beta }^{i}\right)$$

where the sum runs over all central atoms *i*, α is the *i* ^thst^ atom chemical species, β runs over the neighboring chemical species of *i* denoted as the set *B*_*i*_ and *f*_e_ are properly chosen embedding functions. *f*_b_, *f*_a_, *f*_c_, *f*_pw_, and *f*_e_ are the basis functions (potential curves) to be optimized given the current training set. The above representation is a generalized flexible form of a semiempirical FF with an arbitrary shape of the potential curves *f* and direct dependence on the physics aware descriptors (here, pairwise distances, embedding densities, bond distances and bending angles). We should also note that it is quite straightforward to introduce additional terms in the above functional basis set such as neural networks or Gaussian processes that are typically used in ML-based classical FFs.

The embedding part of the model, that is of great importance for the accurate description of many-body interactions, is further clarified below. Each density description of a central atom *i* of type α, ρ_αβ_^*i*^, is calculated by

5 $$\rho_{\alpha \beta }^{i}=\sum_{j\in \beta }\varphi \left(r_{ij}\right)$$

where *j* runs over all neighboring atoms of type β, *r*_*ij*_ is the distance of the atom *j* to the central atom and φ­(*r*) is the Lucy activation function (see Supporting Information Section S1 and Figure S1). Such symmetry function has been employed in coarse grained modeling and they have been implemented in Lammps MD software,^40–42^ however, to the best of our knowledge it is the first time that they are used in atomistic modeling of interfacial systems. We should stress that in the latter model, despite that it accounts for many-body interactions, forces reduce to pairwise summations, and therefore the computational efficiency is preserved.^41,43^ Since it might not be so obvious, taking the derivatives, the force on atom *i*, of type α from embedding contributions of atoms of type β is given by

6 $$F_{i}=-\sum_{j\in \beta }\left[\frac{\partial f_{\alpha \beta }\left(\rho_{\alpha \beta }^{i}\right)}{\partial \rho }+\frac{\partial f_{\beta \alpha }\left(\rho_{\beta \alpha }^{j}\right)}{\partial \rho }\right]\frac{d\varphi \left(r_{ij}\right)}{dr}\frac{r_{i}-r_{j}}{r_{ij}}$$

The first term corresponds to embedding *i* into the atomic environment of β type atoms at positions **r**_*j*_. The second term is the contribution by atom *i* when embedding atom *j* of type β in the atomic environment of α type atoms.

### 2.2 Parameterization

All functions are parametrized partially or completely with Bezier polynomials,^44^ instead of the commonly used cubic splines.^41,45^ Each embedding term is parametrized with a single Bezier curve, while pairwise terms are parametrized via the superposition of a Morse potential and a flexible Bezier curve. Bonds and bond angles are also considered for the covalent interactions in CO, CO_2_ and NO_2_ molecules, using a superposition of a Morse and Bezier curves and a harmonic potential, respectively. The parametrization of the model’s different energetic contributions is summarized below:

7 $$\begin{array}{l} f_{b}\left(r\right)=f_{Morse}\left(r;D_{e},r_{e},\alpha \right)+f_{Bezier}\left(r;k\right)\\ =D_{e}{\left[1-e^{-a\left(r-r_{e}\right)}\right]}^{2}+f_{Bezier}\left(r;k\right) \end{array}$$

8 $$f_{a}\left(\theta \right)=f_{harmonic}\left(\theta ;\theta_{0},k\right)=k{\left(\theta -\theta_{0}\right)}^{2}$$

9 $$\begin{array}{l} f_{pw}\left(r\right)=f_{Morse}\left(r;D_{e},r_{e},\alpha \right)+f_{Bezier}\left(r;k\right)\\ =D_{e}\left[e^{-2a\left(r-r_{e}\right)}-2e^{-a\left(r-r_{0}\right)}\right]+f_{Bezier}\left(r;k\right) \end{array}$$

10 $$f_{e}\left(\rho_{\alpha \beta }\right)=f_{Bezier}\left(\rho_{\alpha \beta };k\right)$$

where terms on the right of “;” denote the training parameters to be optimized. Note that Morse potentials for bonded interactions possess a minimum at zero energy, since bonded interactions describe the internal deformation of the molecule, where the DFT energy is also set to zero at the energetically minimized configuration. Moreover, adding *f*_Bezier_ in the bonded part *f*_b_(*r*), offers more flexibility, which is especially useful when bonded interactions are modified by the presence of other materials in the environment such as Ag atomic clusters (e.g., molecules chemisorbed to the cluster).

Notice that a part of the potential preserves physical awareness, while Bezier curves add the necessary flexibility to capture complex physicochemical interactions. Bezier curves preserve some advantages such as smoothness, continuity, controllability of the shape of the curves with only a few more training parameters (8–13 per curve), compared to the commonly used cubic splines that preserve locality.^39^ However, to our knowledge, this parametrization scheme has never been used before in the development of atomistic models. Detailed information on the Bezier curves and their current implementation is provided in the Supporting Information, while a demonstration of their construction is shown in Figure S2.

From here in, we denote the descriptors by a single vector **d** per structure. Therefore, the total energy of the system might be written as a function of **d** and a parameter set **P**, which defines the shape of the curves.

### 2.3 Model Training

To optimize the classical gas/atomic cluster hybrid model, we consider both the energy loss ( $L_{E}$ ) and the forces loss ( $L_{F}$ ) defined as the mean square error (MSE) over energy and force data derived via DFT calculations per each configuration as

11 $$\begin{array}{l} L_{E}\left(d\left(r\right);P\right)={\left(\mathrm{U̅}\left(d\left(r\right);P\right)-{\mathrm{E̅}}_{dft}\left[r\right]\right)}^{2}\\ L_{F}\left(d\left(r\right);P\right)=\frac{1}{3n_{a}}\sum_{j}^{n_{a}}\;\sum_{k}^{3}{\left({\mathrm{F̅}}^{j,k}\left(d\left(r\right);P\right)-{\mathrm{F̅}}_{dft}^{j,k}\left[r\right]\right)}^{2} \end{array}$$

where **d**, denotes the set of the classical descriptors (e.g., pairwise distances and embedding densities) of the model, which are a function of the configuration **r**, **P** is the set of training parameters, *U* and *E* denote the classical and DFT energies respectively, *F* ^*j*^ is the force on each atom *j*, *k* denotes the direction, *n*_a_ is the number of atoms, and overline symbols denote normalized values such that

12 $$\begin{array}{l} \mathrm{U̅}\left(d\left(r\right);P\right)=\frac{U\left(d\left(r\right);P\right)-\mu_{E}}{\sigma_{E}}\\ {\mathrm{E̅}}_{dft}\left[r\right]=\frac{E_{dft}\left[r\right]-\mu_{E}}{\sigma_{E}}\\ \mathrm{F̅}\left(d\left(r\right);P\right)=\frac{F\left(d\left(r\right);P\right)-\mu_{F}}{\sigma_{F}}\\ {\mathrm{F̅}}_{dft}\left[r\right]=\frac{F_{dft}\left[r\right]-\mu_{F}}{\sigma_{F}} \end{array}$$

where μ_E_, σ_E_ is the mean and standard deviation of the DFT energy data and μ_F_, σ_F_ is the mean and standard deviation of the DFT forces. Denoting D the training set data, the energy cost (*C*_E_), and the force cost (*C*_F_) are defined as

13 $$C_{E}\left(D,P\right))=\frac{1}{n_{d}}\sum_{i}^{n_{d}}{\left(\mathrm{U̅}\left(d\left(r_{i}\right);P\right)-{\mathrm{E̅}}_{dft}\left[r_{i}\right]\right)}^{2}$$

14 $$C_{F}\left(D,P\right))=\frac{1}{3n_{d}n_{a}}\sum_{i}^{n_{d}}\sum_{j}^{n_{a}}\sum_{k}^{3}{\left({\mathrm{F̅}}^{j,k}\left(d\left(r_{i}\right);P\right)-{\mathrm{F̅}}_{dft}^{j,k}\left[r_{i}\right]\right)}^{2}$$

where *n*_d_ is the number of configurations or equivalently data points. To find the optimal set of parameters we initially solve the optimization problem

15 $$P_{0}=\underset{P}{\arg\;\min}C_{E}\left(D,P\right))+\lambda ∥P∥$$

where λ is a constant regularization parameter. Using the initially estimated solution **P**_0_ and eq 15 we compute the $C_{F}^{0}\left(D,P_{0}\right)$ . Then, we iteratively minimize the energy cost at successively lower values of the force cost, by setting an equality constraint for force cost at successively lower values. The overall training algorithm is shown below:
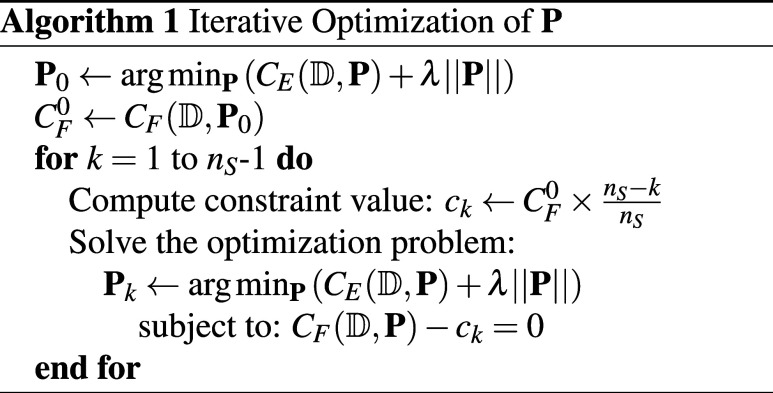


The successive constraint optimization problems are solved via the gradient-based sequential quadratic programming (SQP) method,^46^ starting from 3 randomized initial parameters, in addition to the current best performing solution, either found in the previous AL step or during a current constraint minimization step. Solving for *k* = 0, 1, ..., *n*_S_ – 1 provides respectively, *n*_S_ different solutions, given that the equality constraints can be satisfied. Here we set *n*_S_ = 15. Note that during optimization *C*_E_ and *C*_F_ should in principle be nonconflicting objectives; however, this might not be the case due to noise in the data and the (relatively small) size of the data set. Moreover, considering only energies may result in a solution in the training parameter space that does not necessarily minimize *C*_*F*_, due to overfitting of particular energetic contributions. This is an efficient framework not only to find Pareto optimal solutions but to evaluate different ones. We select the solution that has the best trade-off between the two using the selection metric

16 $$C_{m}=\sqrt{C_{E}^{2}+C_{F}^{2}}$$

Finally, we split the existing data during each AL iteration into a training (train) and a development (dev) set in 80/20%, respectively. The training set is used in algorithm 1, while the development set is used to evaluate *C*_m_ and select the best solution.

### 2.4 Candidate Sampling via Stochastic Methods

The refined FF at each AL iteration is used to generate new candidate structures for labeling. When the initial data set is extremely small (<10 points) 10 new candidates per point are generated by adding a small random Gaussian noise (∼*N*(0, 0.02) in Å) to the initial structures. For early stage models, here between the second up to the 10th AL iterations, candidates are generated via many (here 100) parallel Metropolis–Hastings Monte Carlo (MHMC) simulations, starting from different initial structures, chosen randomly from the existing labeled data. On each step, a random Gaussian perturbation (∼*N*(0, σ)), where σ is autotuned (discussed below), on one randomly selected atom is applied. The proposed (new) structure is accepted as candidate one with probability

17 $$\min\left(1,e^{-b_{a}\left(U\left(d^{new};P\right)-U\left(d^{old};P\right)\right)}\right)$$

where *b*_a_ = 1/*k*_B_ *T*_a_, *k*_B_ is the Boltzmann constant and *T*_a_ is an annealing temperature that becomes higher as we accept new data, following the rule

18 $$T_{a}=T_{s}\;\min\left(1,2\frac{N_{acc}}{N_{tot}}\right)$$

where *T*_s_ is the maximum sampling temperature, *N*_acc_ is the number of accepted structures and *N*_tot_ is the total number of desired candidate structures, here set to 40,000. Hence, half of the structures are sampled at increasing temperature values between 0 and *T*_s_ and half at the maximum sampling temperature. Note that in such small systems temperature is ill-defined; therefore *T*_s_ is an effective hyperparameter that controls the extent of the energy distribution and energy fluctuations.

The reasoning behind the slow increase of *T*_a_ to *T*_s_ is 2-fold: (a) It allows for a smooth scan near PES local minima and (b) can effectively capture transition phenomena that may happen at unknown temperatures, below a hypothesized *T*_s_, like desorption of the gas molecules to the atomic clusters. Such phenomena may be very fast at high effective temperatures and extremely slow at low ones, where in both cases information on the path of this structural rearrangement may be lost. Slowly increasing *T*_a_ allows for smoother transitions.

Finally, the step length σ is autotuned such that the acceptance ratio (AR) remains between 0.2 and 0.5. It is not allowed to exceed 0.2 Å and does not go below 0.001 Å. If the running mean of the AR is below 0.2 σ, it is multiplied by 0.99, while if it is above 0.5, it is divided by 0.99. The initial σ is set to 0.02 Å. Moreover, the first 100 MC steps are used for equilibration and therefore no structures are accepted. This MC sampling procedure ends when *N*_acc_ ≥ *N*_tot_.

For later stage models, after the 10th AL iteration, stochastic molecular dynamics, sMD, (Langevin dynamics) simulations are used starting from one initial configuration. The system runs for 2 ns, with a time step of 0.1 fs to avoid plausible numerical instabilities, especially of early stage models. The damping factor used to calculate the friction forces as described by ref 47 was set to 1 fs. Structures are sampled every 50 fs, resulting in 40,000 candidate structures. In contrast to MC sampling, we chose just one initial configuration from the existing data set, which is energetically minimized. In the first 1 ns, we anneal the temperature from 1 K to *T*_s_ (here, set to 500 K) , similarly to the MC sampling procedure. sMD is performed using the open source software LAMMPS.^40^

### 2.5 Data Selection & Outlier Score

Next, our goal is to select, from the above pool of many (here 40,000) candidate structures, a relatively small subset (here we use a predefined batch of *n*_batch_ = 200 structures) for DFT labeling. The selected configurations are used to evaluate the performance of the model and at the same time enrich our data set for the next iteration of the AL-based optimization scheme. For this, we use a metric of the variability of the candidate structures with respect to the structures in the existing data set. In more detail, the selection probability of each structure is proportional to its computed outlier score (OS) which is calculated as follows:

For each element *d* in the descriptor vector $d={\left[d_{1},d_{2},...,d_{m}\right]}^{T}$ of a given (candidate) structure, we compute a distribution overlap parameter

19 $$\omega \left(d\right)=\int_{h_{low}}^{h_{up}}h\left(x\right)e^{-a{\left(x-d\right)}^{2}}dx$$

where *h*(*x*) represents the histogram density of the descriptor element *d* (which is also an estimate of the probability distribution function) in the existing data set, *x* is an integration parameter, *h*_low_ and *h*_up_ denote the lower and upper bounds of the distribution, respectively, and *a* is a scaling parameter set as *a* = *h*_up_ – *h*_low_. For example, if *d* represents a specific pairwise atomic distance in a candidate structure, then *h*(*x*) denotes the probability distribution function (PDF) of this distance in the existing data set. The overlap of the distribution ω­(*d*) is effectively a smooth approximation of the density of the histogram at *d* and practically a measure of the variability of a new candidate configuration with respect to the underlying probability distributions of each element in the descriptor vector. This value is normalized as

$$\mathrm{\omega ̅}\left(d\right)=\frac{\omega \left(d\right)-\omega_{\min}}{\omega_{\max}-\omega_{\min}}$$

where

20 $$\omega_{\min}=\min_{z}\left(\int_{h_{low}}^{h_{up}}h\left(x\right)e^{-a{\left(x-z\right)}^{2}}dx\right)\;\forall z\in \left[h_{low},h_{up}\right]$$

21 $$\omega_{\max}=\max_{z}\left(\int_{h_{low}}^{h_{up}}h\left(x\right)e^{-a{\left(x-z\right)}^{2}}dx\right)\;\forall z\in \left[h_{low},h_{up}\right]$$

Note that, here, *z* is just a minimization or maximization parameter and ω_min_ and ω_max_ depend only on the distribution *h*(*x*). ω̅(*d*) takes values in [0,1], when *d* is within the range of the distribution [*h*_low_, *h*_up_] and can take negative values if ω­(*d*) < ω_min_, which denotes out of distribution data. Especially at the early stages of the AL iteration where *h*(*x*) is very narrow and ω_max_ – ω_min_ is small ω̅(*d*) may take highly negative values.

In the case of a one-dimensional descriptor vector, selecting data with a probability of 1 – ω̅(*d*) favors out-of-distribution or rare data points. Therefore, the outlier score OS is computed by

22 OS(d)=1−ω̅(d)

and each new candidate is selected via a probability

23 $$s\propto \frac{OS-OS_{\min}}{OS_{\max}-OS_{\min}}$$

where OS_min_ and OS_max_ are the minimum and maximum OS values in the set of the current candidates. The latter normalization ensures that the selection probability is much higher for the relatively more deviating candidates. Extended discussion and example distributions of *h*(*x*) and their corresponding OS computation is provided in Figure S3 in the Supporting Information.

For a multidimensional descriptor vector $d\in R^{m}$ , as is the case in this work,

24 $$OS\left(d\right)={\left(\frac{1}{m}\sum_{i=1}^{m}{\left(1-\mathrm{\omega ̅}\left(d_{i}\right)\right)}^{3}\right)}^{1/3}$$

where the power mean is used to emphasize larger values of 1 – ω̅(*d*), ensuring that rare or out-of-distribution descriptor values contribute more to the OS.

### 2.6 Data Labeling & Performance Evaluation

In the above framework *E*_dft_ and *F*_dft_ denote the labeled energy and forces calculated from DFT. The absolute value of the energy calculated at the DFT level should be modified according to the nature of the underlying interactions embodied in the form of *U*(**r**). For the case of isolated atomic metal nanoclusters the energy is *E*_dft_ ≔ *E*_coh_, where coh denotes cohesive interactions, so that *U*_coh_(**r**) ≈ *E*_coh_[**r**] for all plausible configurations **r**. For the notation below let the subscript denote the nature of the interaction and the superscript the structures involved. Let cl and gas denote the structure of the cluster and gas, respectively. No subscript denotes the total energy of the system. Then, the cohesive energy for the cluster is given by

25 $$E_{coh}^{cl}\left[r^{cl}\right]=E^{cl}\left[r^{cl}\right]-\sum_{t}n_{t}E_{t}^{1\;atom}\left[r^{1\;atom}\right]$$

where t denotes the different atom types, *n*_*t*_ the number of atoms of that type and *E*_t_^1atom^[**r** ^1atom^] is the energy of one atom of type t. The cluster deformation energy is given by

26 $$E_{\mathrm{def}}^{cl}\left[r^{cl}\right]=E^{cl}\left[r^{cl}\right]-E_{\min}^{cl}\left[r_{\min}^{cl}\right]$$

where min denotes the minimum total energy and the corresponding structure. Similarly for the gas

27 $$E_{\mathrm{def}}^{gas}\left[r^{gas}\right]=E^{gas}\left[r^{gas}\right]-E_{\min}^{gas}\left[r_{\min}^{gas}\right]$$

Now, let the interaction energy in the cluster/gas complex (cg)

28 $$E_{\mathrm{int}}^{cg}\left[r^{cg}\right]=E_{ads}^{cg}\left[r^{cg}\right]-E_{\mathrm{def}}^{cg}\left[r^{cg}\right]$$

where

29 $$E_{ads}^{cg}\left[r^{cg}\right]=E^{cg}\left[r^{cg}\right]-E_{\min}^{cl}\left[r_{\min}^{cl}\right]-E_{\min}^{gas}\left[r_{\min}^{gas}\right]$$

and

30 $$E_{\mathrm{def}}^{cg}\left[r^{cg}\right]=E_{\mathrm{def}}^{cl}\left[r^{cl}\right]+E_{\mathrm{def}}^{gas}\left[r^{gas}\right]$$

Plugging eqs 29, 30, 26 and 25 into [Disp-formula eq28] and after elimination of terms

31 $$\begin{array}{l} E_{\mathrm{int}}^{cg}\left[r^{cg}\right]=E^{cg}\left[r^{cg}\right]-E_{\min}^{gas}\left[r_{\min}^{gas}\right]-E_{\mathrm{def}}^{gas}\left[r^{gas}\right]-E_{coh}^{cl}\left[r^{cl}\right]\\ -\sum_{t}n_{t}E_{t}^{1\;atom}\left[r^{1\;atom}\right] \end{array}$$

which may be rearranged as

32 $$\begin{array}{l} E_{\mathrm{int}}^{cg}\left[r^{cg}\right]+E_{\mathrm{def}}^{gas}\left[r^{gas}\right]+E_{coh}^{cl}\left[r^{cl}\right]=E^{cg}\left[r^{cg}\right]\\ -\left(E_{\min}^{gas}\left[r_{\min}^{gas}\right]\left[r^{cg}\right]+\sum_{t}n_{t}E_{t}^{1\;atom}\left[r^{1\;atom}\right]\right) \end{array}$$

The latter form is particularly convenient. First, for each data point, only the total energy of the complex *E* ^cg^[**r** ^cg^] must be calculated since the remaining terms on the right-hand side of the equation are calculated only once and their sum is denoted by *E*_ref_. Most importantly, it allows one to tackle all the interactions simultaneously by assuming that

33 $$U_{\mathrm{int}}^{cg}\left(r^{cg}\right)+U_{\mathrm{def}}^{gas}\left(r^{gas}\right)+U_{coh}^{cl}\left(r^{cl}\right)\approx E^{cg}\left[r^{cg}\right]-E_{ref}≔E_{dft}$$

The above formulation assumes that the cluster/gas interaction, the gas deformation, and cluster cohesive interactions are decoupled. Hence, it allows tackling the cluster/gas system together with their isolated cluster and gas counterparts, augmenting the data set in the sense that we effectively label the energy of the cluster/gas system at well-separated distances. This is particularly important for learning the different energetic contributions of eq 33.

#### 2.6.1 Density Functional Theory Settings

All DFT calculations were performed using the Gaussian software.^48^ with ωB97XD hybrid functional and the def2SVP basis set.

#### 2.6.2 Performance Metric

After we label the new structures with DFT energies, we evaluate the performance of the new model via the mean absolute error (MAE), defined as

34 $$MAE\left(D,P\right)=\frac{1}{n_{d}}\sum_{i}^{n_{d}}\left|U\left(d\left(r_{i}\right);P\right)-E_{dft}\left[r_{i}\right]\right|$$

Note that this metric is used for the evaluation of the model and not during the training; for the latter, the iterative optimization scheme, including energies and forces, is used.

### 2.7 Active Learning Initialization and Stopping Criterion

The AL procedure may start by using one or more configurations from one system or a collection of systems. If a collection of systems is used, then the algorithm will assume different interactions based on the type of chemical elements. For example, including in the initial data set Ag clusters of different sizes, the algorithm will train one model describing interactions between Ag atoms for all the different cluster sizes. All initial structures were obtained from the work of Mohammadi et al.^35^ and were reoptimized using the current DFT settings.

The AL algorithm was terminated when both the prediction error and the outlier score exhibit quasi-constant values for at least 5 consecutive AL iterations while sMD was used for sampling (after the 10th AL iteration).

### 2.8 Code

The code, written mainly in Python 3 and bash, is a package of scripts that automatically communicates with the high-performance facility scheduler SLURM and, respectively, performs the DFT calculations in parallel using the Gaussian software. Similarly, it automatically communicates with LAMMPS. The code takes as input one single input file, which includes the settings and an initialization of the model parameters. All model parameters may be considered as fixed or training parameters, allowing for transfer learning options.

### 2.9 Single Ag Cluster in Bulk Gas Phase MD Simulations

The FFs produced for Ag/gas (Ag_7_CO_2_, Ag_8_CO and Ag_4_SO_2_) were utilized in the bulk gas phase. To create respective bulk phase systems, we performed the following steps: First, for each of these 3 cases, 1000 gas molecules were created in a simple cubic lattice with a lattice constant suitable to reproduce the gas phase density of CO_2_, CO and SO_2_ gases under ambient conditions. Then, a single Ag_7_, Ag_8_ and Ag_4_ configurations was inserted in the middle of the simulation box, for each case, respectively. The time step was set to 1 fs. Random initial velocities corresponding to 300 K were used. Langevin dynamics were performed with a damping factor set to 10 fs using LAMMPS as done during the sampling step of AL. Gas/gas nonbonded interactions were modeled using the COMPASS FF.^49^ A cutoff radius of 2 nm was used.

## 3 Results and Discussion

### 3.1 Application to the Ag_7_CO_2_ Hybrid

We apply the AL algorithm to a diverse range of systems characterized by varying physicochemical interactions, demonstrating the broad applicability of our approach. These systems include undercoordinated Ag clusters governed by cohesive interactions, due to metallic bonds, as well as Ag/gas atomic cluster hybrids, where both strong physisorption and chemisorption interactions complicate the PES. Note also that models developed in the bulk phase, such as EAM,^11^ are not suitable, exactly due to the heterogeneous coordination of these systems. In addition, we explore different cluster sizes to assess the robustness of the algorithm. To illustrate its effectiveness and behavior, we first employ the method to actively optimize the FF describing the interactions within the Ag_7_CO_2_ hybrid (comprising of 7 Ag atoms and one CO_2_ molecule). We then validate its general applicability and transferability in Section 3.2.

Throughout this work, we assume that the covalent interactions of the gas molecule, the cohesive forces among the metallic atoms, and the gas/metal interactions are decoupled (see eq 33). Consequently, the cohesive metallic interactions and the covalent interactions in the gas molecule can, in principle, be optimized independently of the metal/gas ones. Indeed, we use the AL algorithm to optimize the potential describing the covalent interactions of the isolated CO_2_ molecule. Due to its simplicity, six AL iterations with *n*_batch_ = 50 were enough and the prediction error was about 1.0 meV/atom. The respective potential terms are kept fixed during the training of the gas/metal FF. In contrast, the cohesive forces among the Ag atoms are optimized simultaneously with the gas/metal interactions. We initialize the algorithm with both the isolated Ag_7_ cluster and the Ag_7_CO_2_ hybrid. By generating and augmenting with data of the isolated cluster, and fixing the covalent potential terms optimized for isolated CO_2_, we effectively “encourage” the model to learn the energies of well separated systems (the gas being far away from the metal cluster) and, hence, the true energetic contributions (e.g., cohesive, gas physisorption) to the total energy.

Note that in this framework, we label the DFT energy as the sum of all the interactions in each system (see Section 2.6). Labeling the metal/gas interaction energy explicitly would demand, for each new structure of the hybrid, to compute the DFT energies of the deformed Ag_7_ cluster and CO_2_ molecule in an isolated environment. Moreover, in multigas systems such an alternative would demand to isolate each different gas molecule and to perform respective single point DFT calculations, which further increases computational costs. Although in some interfaces this deformation energy was assumed to be negligible in the past,^34^ here, the undercoordinated clusters deform significantly, and so are the gases due to the strong interactions, especially for SO_2_ chemisorption.

The total training, development and prediction MAEs of the derived models, trained on data from both isolated Ag_7_ and Ag_7_CO_2_ structures, as a function of the AL iterations are shown in Figure 2a. During the first AL iteration the FF is trained on just one data point per system. Due to that very initial small data set, in the first iteration 20 new candidates (10 per system) are generated by adding a small random Gaussian noise (∼*N*(0, 0.02) in Å) to the 2 initial structures. Consequently, the prediction error seems small. Next, during the second up to the 10th AL iteration, new candidate structures are generated by MHMC, while for the consequent AL iterations (from the 11th up to the last one), sMD is performed, as described in the Methods Section 2.4. Initial steps show very low training and development MAE; however, this is a result of overfitting to the small initial data set and the restricted exploration of the PES, giving rise to high prediction MAE. Later, the training/development set error is approximately constant at about 30 meV, indicating the model’s capacity. Switching to sMD at the 11th iteration results in a higher prediction error, especially for the hybrid Ag_7_CO_2_ system, as shown in Figure 2b. As the AL algorithm explores new regions of the PES, it is likely to show increased prediction error, such as cases where the molecule desorbs or reorients in a different way near the Ag cluster. This increase indicates that sMD explores new regions of the PES more effectively.

**Figure 2:**
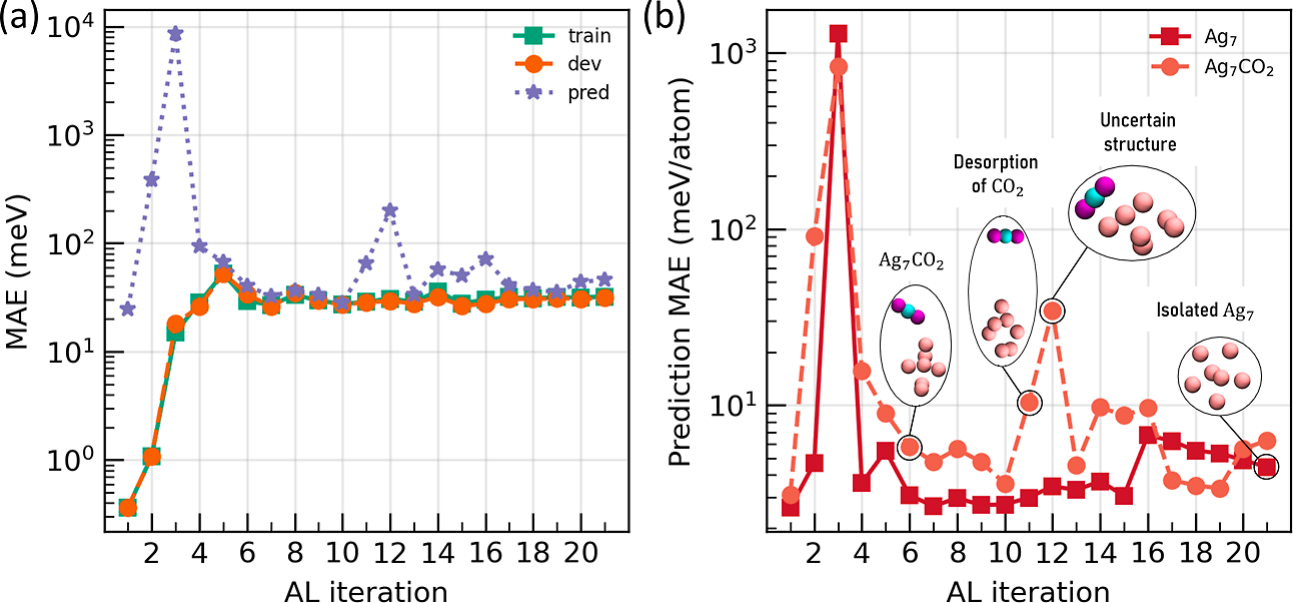
(a) Training (train), development (dev) and prediction (pred) MAE (meV) of the derived models, of the Ag_7_CO_2_ hybrid, as a function of the AL iterations. (b) Prediction MAE in energies (meV/atom) for each system were the FF is actively trained simultaneously, as a function of AL iteration. Indicative structures are depicted. At the 11th iteration sMD resulted in desorption of the CO_2_ molecule, while in the 12th sMD drove the system in unexplored regions of the PES, increasing the error.

Evidently, the selected structures, from the pool of candidates generated from sMD (after the 10th AL iteration), exhibit higher outlier scores (OS, defined in eq 24) as depicted in Figure 3. This Figure depicts the mean, standard deviation, maximum, and minimum values of the OS for the selected batch at each AL iteration, defined respectively as $\overset{®}{OS}$ , OS_std_, OS_max_, and OS_min_, for the sake of discussion. By definition, OS­(**d**) > 1 denotes that, with respect to the specific descriptor **d**, the candidate structure is outside of the distribution of **d** in the existing data set, while values close to one denote a structure that is dissimilar to the majority of the structures in the existing data set. In the first few (two-three) iterations, especially for Ag_7_CO_2_, the existing data distributions for each descriptor element are quite narrow, resulting in elevated $\overset{®}{OS}$ and OS_max_. In later iterations $\overset{®}{OS}$ is near 0.4. Between the fourth and the 10th AL iteration, OS_max_ stays below 1, while during sMD, it takes values above 1 and the $\overset{®}{OS}$ is higher, indicating exploration of new PES regions. Toward the last AL iterations, the $\overset{®}{OS}$ converges to a value of about 0.45 indicating that the underlying descriptor distributions in the existing data are converging. Moreover, the oscillations in $\overset{®}{OS}$ seem to correlate well with the oscillations in the MAE prediction of Figure 2b. In addition, the differences in $\overset{®}{OS}$ between the isolated cluster systems and the gas hybrid demonstrate the much more complex PES of the hybrid system. Despite that, after 21 iterations, the AL seems to have explored well the PES, with prediction MAE below 6 meV/atom. This is a significant result, considering that a superposition of flexible physics-aware models is used as the functional basis set, that preserves pairwise additive computational efficiency (computational cost scales with *N* log *N*, using cell link lists, where *N* is the number of atoms).

**Figure 3:**
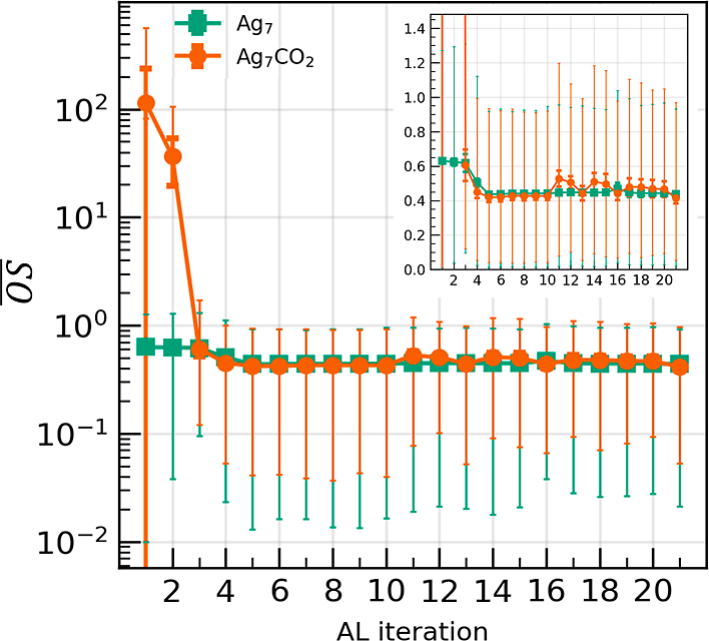
Mean outlier score, $\overset{®}{OS}$ , for the selected batch at each AL iteration, per system. Thick caps denote standard deviation, while thin caps denote the maximum and minimum values. In the inset the data are shown in a linear plot.

Training the FF for such systems is also a challenging task. Finding the parameter set that globally minimizes the cost function (13) is not trivial, especially for multiparametric models with strong nonlinear dependencies and interfacial PES.^34^ Here, we select the solution that ensures the best trade-off between the energy cost (*C*_E_, eq 13) and the forces cost (*C*_F_, eq 14) among the Pareto optimal solutions. Pareto optimal solutions are defined as the ones for which there is no other solution that is better for both objectives. As described in the Methods section, these are found by initially minimizing the *C*_E_ without considering *C*_F_, and then minimizing *C*_E_ while constraining the *C*_F_ at subsequently descending values. Here, indicatively, we choose to show the set of calculated solutions during the 13th AL iteration in Figure 4. Not all solutions are Pareto optimal, including the initial one, due to the various different parameter sets that locally minimize *C*_E_. Statistical differences between the training and the development set might also affect this behavior. Preeminently, this training approach provides a way to evaluate different model solutions and choose the solution among them exhibiting the best trade-off between *C*_E_ and *C*_F_. In the inset, we demonstrate the agreement of the “best” solution with DFT forces. The positive effect of this methodology is even more pronounced in premature models in the early stages of AL, as discussed in the Supporting Information Section S4 and shown in Figure S4. Finally, the model derived parameters at each AL iteration are shown in Figure S5. Toward the last AL iterations, these exhibit convergence, and therefore the model converges to a similar functional form. Overall, this constrained optimization strategy, unlike the typical joint loss approach (*L* = *C*_E_ + λ*C*_F_), ensures robustness and leads to more stable and physically meaningful FFs in low-data regimes, also avoiding tuning hyper parameters like λ in the joint loss.

**Figure 4:**
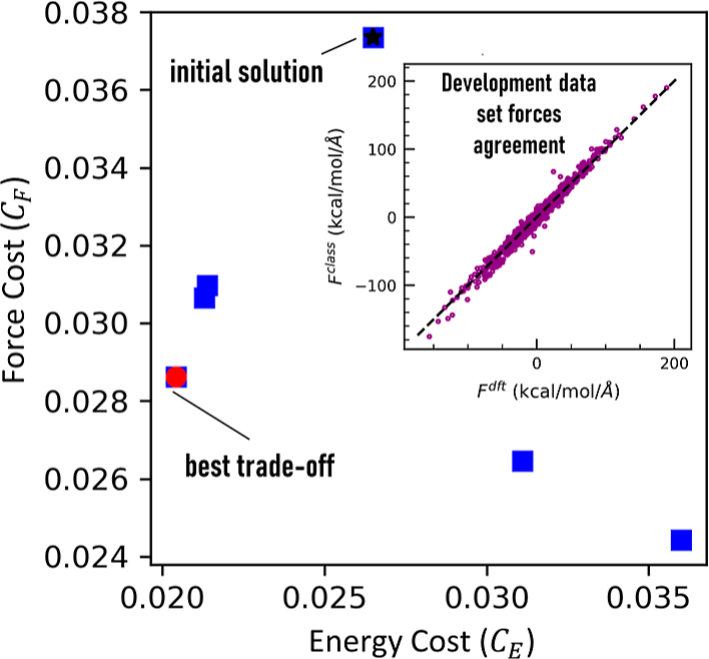
Set of calculated solutions (blue squares) on the normalized force (*C*_F_) and energy cost (*C*_E_) for the development set during the 13th AL iteration. Note that not all solutions are Pareto optimal, including the initial solution (black star), where we do not include the forces in the training. The solution that exhibits the best trade-off (red circle) of these two error metrics in the development set is kept. In the inset, we show the classical model’s agreement with DFT forces. Since *C*_F_ and *C*_E_ are normalized quantities they are dimensionless.

### 3.2 General Applicability & Transferability

Besides the above demonstration case, we applied the algorithm in several additional systems; here we present results obtained from two more gas/atomic cluster hybrids and we consider several Ag cluster sizes on an additional third case, as summarized in Table 1. The second system (case 2) concerns a hybrid of an Ag cluster with different size compared to the first one, and the CO gas. CO exhibits stronger physisorption interactions with Ag, compared to the CO_2_. The hybrid in case 3 involves SO_2_ and the planar atomic cluster Ag_4_. The planarity of the cluster, the exchange of electrons between SO_2_ and Ag (chemisorption characteristics) and the strong SO_2_/Ag interactions result in an even more complex PES of the hybrid. To fit well the different energetic contributions, we optimize the FF simultaneously for all energy terms (gas covalent, cluster cohesive, and hybrid chemisorption) and explore via the AL procedure the PES for the Ag_4_SO_2_ hybrid and both the isolated Ag_4_ and *SO*_2_. Due to the PES roughness, we sampled for a larger number of AL iterations. Case 4 involves Ag clusters of multiple sizes, while the FF is common for all systems (Ag atoms are treated as the same type).

**Table 1:** Different Applications of the AL Algorithm^a^

case	systems	characteristic interactions	energy perf. (meV/atom)	force perf. (meV/Å)	AL Iters.
1	Ag_7_, Ag_7_CO_2_	cohesive, physisorption	5.3, 4.3	62, 77	21
2	Ag_8_, Ag_8_CO	cohesive, physisorption	6.6, 6.7	88, 89	21
3	Ag_4_, Ag_4_SO_2_, SO_2_	cohesive, chemisorption	12.3, 22.7, 2.2	122, 145, 78	65
4	Ag_9_, Ag_10_, Ag_11_, Ag_12_	cohesive	17.5, 34.3, 23.2, 31.3	154, 168, 135, 144	21
	Ag_13_, Ag_14_, Ag_15_, Ag_16_		27.0, 21.6, 11.1, 15.2	162, 191, 141, 161	
5	Ag_17_, Ag_18_	cohesive	12.8, 8.6	126, 149	6

a The energy and force prediction performance is averaged over the last 5 AL iterations, shown in order respective to the naming order in the systems column. In each AL iteration, 200 new structures are evaluated, distributed equally among all systems (i.e., each system receives an equal number of data points).

Figures S6–S8 present the predicted MAE and selected batch OS statistics as a function of AL iterations for cases 2–4, respectively. A similar convergence trend is observed across these cases, with the OS correlating well with the predicted MAE. In Case 2, after 21 AL iterations, the model achieves excellent accuracy, with an MAE below 10 meV/atom. In Case 3, the model successfully captures the complex chemisorption interactions, with an MAE of approximately 30 meV/atom. This level of error is acceptable given the model’s simplicity and computational efficiency. Case 4 demonstrates that a unified FF model for a range of cluster sizes (9–16 atoms) can be developed, balancing accuracy and generalizability with an MAE between 10 and 35 meV/atom. All relevant details on the derived models and their optimized parameters are provided in Tables S1–S4 for cases 1–4, respectively.

To assess the transferability across the cluster size, we use the FF optimized at the last AL iteration of case 4 to generate a batch of 200 DFT labeled data of the Ag_17_ and Ag_18_ atomic clusters, that were not included in the training set. Here, the candidate structures are produced using sMD for all AL iterations. The prediction MAE for the first batch is 19.3 and 14.3 meV/atom, for Ag_17_ and Ag_18_ respectively, indicating that the FF is transferable for larger cluster sizes. In addition, we use the AL algorithm to refine part of the FF, specifically for these two cluster sizes. Without increasing the number of descriptors, we include a potential energy term, described via a Bezier polynomial, that depends on the embedding density, up to embedding densities of value 6 (undercoordinated atoms). The part of the potential that was optimized in case 4 is kept fixed, while we fine-tune this new potential term. We run 6 AL iterations and the results are shown in Figure 5. The prediction MAE drops even below 10 meV/atom. This is a “transfer learning” procedure and, although, here, we have not increased the complexity of the model, for example using sophisticated many-body descriptors, such as the SOAP,^15^ we demonstrate that such smooth potentials could learn most of the essential physics, improving transferability and extrapolation of the model.

**Figure 5:**
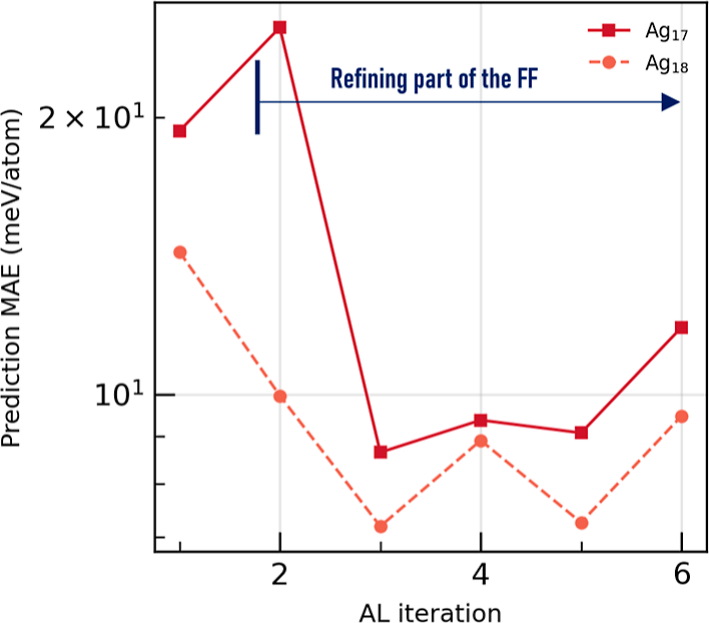
Prediction MAE for Ag_17_ and Ag_18_ as a function of AL iteration. In the first iteration, 200 data points are generated and predicted via the FF optimized in case 4. Then, part of this FF is fined tuned to the specific data. On each iteration 200 new data are generated.

Figures S9–S13 depict the underlying energy, energy fitting error and descriptor distributions for cases 1 to 5, respectively. Evidently, the descriptor distributions of case 4 and case 5 are different, however, the range of the descriptor distributions in case 5 is narrower (ρ values reach 10 in case 4 while for case 5 reach up to 8). Larger clusters may exhibit ρ values up to about 12 (coordination of bulk Ag atoms). Since the model learns the underlying physical trends it may extrapolate to even larger clusters, however, confirming this would require additional DFT calculations, which were not performed due to their high computational cost. The model should consist of at least a good starting point for FF development of bulky clusters. The refined potential parameters are provided in Table S5, while the refined potential is shown in Figure S15.

Force errors, as shown in Table 1, follow numerical trends similar to those of the corresponding energy errors. Their absolute values are approximately 1 order of magnitude larger, as expected due to the differing physical units of energy (meV/atom) and forces (meV/Å). This suggests that our model does not significantly overfit any specific contribution to the system’s total energy. To confirm this, we compute the different energy components defined in (33), namely *U*_int_^cg^, *U*_def_^gas^, and *U*_*coh*_^*cl*^, along with their respective DFT energies, *E*_int_^cg^, *E*_def_^gas^, and *E*_coh_^cl^. This is done by isolating the gas and cluster structures from 200 randomly selected hybrid systems and computing their DFT energies, as described in Section 2.6. The corresponding errors for the three gas/cluster systems are reported in Table 2. In all cases, the total energy error exceeds the individual component errors, demonstrating that the model does not simply distribute the total error uniformly among the different contributions. Individual contribution errors are bounded by the total error. To a small extent the decoupling approximation seems to fail, but this is expected since at interfaces interactions are of many-body character, while the energy contributions of this model consider only 2 element types each.

**Table 2:** Error Estimates on the Different Energy Contributions in Hybrid Systems

case	system	total MAE (meV/atom)	cohesive MAE (meV/atom)	gas deformation MAE (meV/atom)	gas/cluster interaction MAE (meV/atom)
1	Ag_7_CO_2_	4.3	2.9	0.2	2.9
2	Ag_8_CO	7.7	6.1	0.008	5.9
3	Ag_4_SO_2_	26.7	26.5	1.9	17.1

### 3.3 Single Ag Clusters in Bulk Gas Phase MD Simulations

To demonstrate the applicability and scalability of our approach, we applied the FFs developed for cases 1, 2, and 3 to respective bulk gas phase systems embedded with a single Ag cluster as described in the methods Section 2.9. We refer to these systems as bulk-CO_2_/Ag_7_, bulk-CO/Ag_8_ and bulk-SO_2_/Ag_4_, respectivily. Systems are left to run for several ns, demonstrating the stability of the FFs and their computational efficiency. Moreover, for some systems (bulk-CO/Ag_8_ and bulk-SO_2_/Ag_4_) a droplet formation ignited by the cluster is observed. This is presented in Figure 6, which shows the nucleation kinetics around the respective Ag cluster. These kinetics are quantified using the autocorrelation function

35 $$K\left(t\right)=\left\langle a\left(t+\tau_{0}\right)a\left(t\right)\right\rangle$$

where *a*(*t*) is 0 if the gas molecule is not condensed into the droplet, and 1 otherwise. Average is over multiple gases and multiple time origins τ_0_. A gas molecule is considered to be within the droplet (*a*(*t*) = 0) if either of its atoms is within 0.35 nm of another atom in the droplet or one of the Ag atoms.

**Figure 6:**
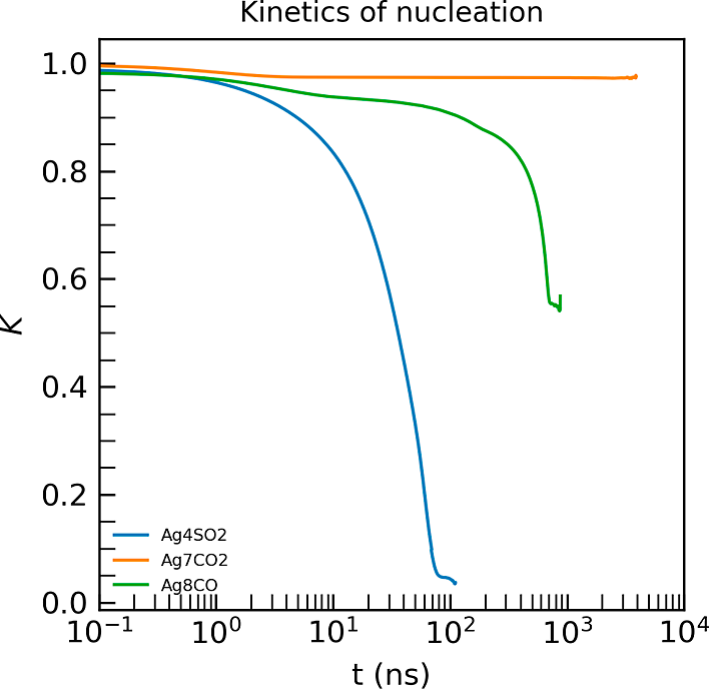
Kinetics of nucleation for bulk-CO_2_/Ag_7_, bulk-CO/Ag_8_ and bulk-SO_2_/Ag_4_ systems.

Droplet formation is initiated on time scales shorter than 10 ns for the SO_2_ gas phase and around 200 ns for the CO gas phase. CO_2_ molecules, however, do not exhibit any condensation behavior within the simulation window of about 4 μs. This sharp phase transition is triggered by the formation of the first adsorbed layer, which generates long-range electric fields. These fields attract strongly or weakly polar molecules, like SO_2_ and CO, respectively, thereby enhancing droplet growth and reinforcing the electric field itself. Nevertheless, entropic effects also play a significant role. Although CO_2_ is more polar than CO, it does not condense, suggesting that polarity alone is not sufficient for droplet formation. Differences in cluster size and Ag-gas interactions may also influence this behavior, but a detailed analysis lies beyond the scope of this study. These results illustrate that the developed models are well-suited for probing key properties relevant to gas sensing applications, such as condensation dynamics and cluster-mediated nucleation in realistic conditions.

## 4 Conclusions

In this work, a batch active learning (AL) framework has been developed and successfully applied to complex gas and atom-sized metal cluster hybrids. The AL algorithm iteratively trains and refines physics-aware FFs. On each AL step, the refined FF is used to generate a large pool of candidate structures via stochastic methods, i.e., MHMC and sMD. The temperature is annealed from 1 K to a high value, *T*_s_ (set to 500 K here), to smoothly capture plausible desorption or reorientation of gas molecules around the rough atom-sized silver (Ag) clusters, thereby exploring the PES. For each candidate structure, an OS is calculated that respects the permutational, rotational, and translational invariance, as well as the permutational invariance of the descriptor vector. The AL algorithm then selects a small batch from the candidates with a probability proportional to their relative OS, efficiently augmenting with new regions of the PES since the selected structures undergo density functional theory (DFT) labeling, and augment the training data set in the next AL iteration. We should emphasize that, here, we iteratively generate the training data set, in contrast to other AL approaches that strategically select data from a predefined labeled pool.^32^ This consists of a great challenge, since a poor model could result in poor augmentation of the data set.

The FFs’ basis set is composed of two-body and many-body terms (and covalent if necessary), similar to semiempirical potentials, while maintaining the computational efficiency of pairwise additive interactions. The model relies on physically driven descriptors, each independently contributing to the total potential energy and predicts well the interactions of complex gas/metal atomic size hybrids. It can capture complex interactions with high accuracy, such as strong physisorption on rough surfaces and cohesive forces in undercoordinated atomic clusters (Ag_7_CO_2_, Ag_8_CO). With an acceptable trade-off in accuracy, a FF describing complex chemisorption interactions for the Ag_4_SO_2_ hybrid is also derived. Furthermore, with a similar trade-off in accuracy, a transferable FF across the cluster size (Ag_9–18_) is produced. Despite the above, the current FF cannot describe reactions such as more advanced machine learning potentials.^4,25^

In this work, we demonstrate that AL approaches can be effectively combined with low-cost models, by generalizing the form of semiempirical potentials and combining them with the smooth, flexible, and controllable Bezier polynomials. As demonstrated, such models can be used either directly for quantitative MD simulations of larger bulk gas phase systems, examining properties like condensation dynamics, leading to sensing insights. Moreover, these models consist a starting point for further optimization on extended functional basis sets, akin to the “transfer learning” procedure followed in Section 3.2. For example, it is highly plausible that a small NN, which takes as input intuitive symmetry functions^14^ will effortlessly fit the deviation of such physics-aware models, instead of trying to directly predict the PES. The logic is similar to the work of Deringer et al.,^21^ who showed that ML models can better extrapolate when physics aware 2- and 3-body terms are used. However, we leave the examination of this hypothesis for future work.

## Supplementary Material



## Data Availability

Data generated in this study are openly available in https://zenodo.org/records/15543078, while code and input files for reproduction of the above results are openly available in https://github.com/SimEA-ERA/Active-Learning-Package.

## Notes

Embedding density activation function, Bezier parameterization, outlier score computation; discussion on the training method, general applicability figures, optimized force field parameter tables, energy, energy fitting error and descriptor distributions, optimized potential plot for case 5 (PDF)
