# Supplementary material for: Active Learning of Atomic Size Gas/Solid Potential Energy Surfaces via Physics Aware Models
Source: J Chem Inf Model. 2025 Aug 12;65(17):9009–21. doi: 10.1021/acs.jcim.5c01193 (PMC12421685; doi:10.1021/acs.jcim.5c01193)
Supplement: Supplementary file 1 [file ci5c01193_si_001.pdf]

# Supporting Information: Active Learning of Atomic Size Gas/Solid Potential Energy Surfaces via Physics Aware Models

Nikolaos Patsalidis ,[†] Mohsen Doust Mohammadi,[†] Somnath Bhowmick,[†] George Biskos,[†,‡] and Vagelis Harmandaris[*,†,‡,¶]

†*Computation-based Science and Technology Research Center, The Cyprus Institute, 2121, Cyprus*

‡*Department of Mathematics and Applied Mathematics, University of Crete, Heraklion, GR-71110, Greece*

¶*Institute of Applied and Computational Mathematics, Foundation for Research and Technology - Hellas, Heraklion, GR-71110 Crete, Greece*

E-mail: v.harmandaris@cyi.ac.cy

## S1 Embedding Density Activation Function

The Lucy activation function defined below:

$$\varphi(r) = \begin{cases} 1 & \text{for } r < r_0 \\ c_0 + c_2 r^2 + c_4 r^4 + c_6 r^6 & \text{for } r_0 \le r \le r_c \\ 0 & \text{for } r > r_c \end{cases} \tag{S1}$$

is depicted in Figure S1. The coefficients $c_0$, $c_2$, $c_4$, and $c_6$ are determined by imposing continuity of $\varphi$ and its first derivatives at $r_c$ and $r_0$, here set to 0.1 and 5.5, respectively. These are considered as hyperparameters of the model, as the form of the activation function can be altered as shown in Figure S1.

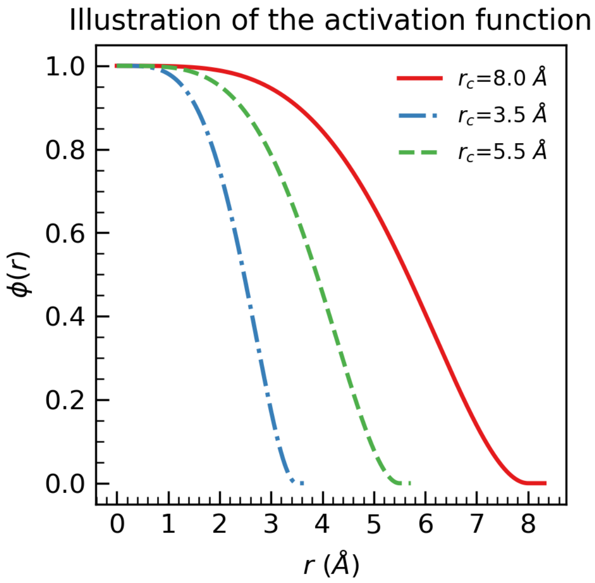


Figure S1: Illustration of the Lucy activation function used to calculate the embedding densities, for different hyperparameter $r_c$. Here $r_0$ is set to 0.1. Models developed in this work used $r_c = 5.5$ Å.

## S2 Bezier Parameterization

The 2D Bezier curve is parameterized by a set of $(b_x^m, b_y^m)$, $m = 0, \ldots, N_b$ points, called control points (CPs). $N_b$ defines the degree of the Bezier polynomial. This a parametric polynomial in the form of

$$x = x(t, b_x^0, \ldots, b_x^{N_b})$$
$$y = y(t, b_y^0, \ldots, b_y^{N_b}) \tag{S2}$$

where $t \in [0,1]$. Each curve begins and ends to the point $(b_x^0, b_y^0)$ and $(b_x^{N_b}, b_y^{N_b})$ respectively. Moreover, the first and second points define the derivative at the beginning of the curve and so are the last two points at the curve end. The first three points define the second derivative at the curve starting point, and so forth. This is a very convenient feature, since we can control the smoothness and continuity of the derivatives at the curve edges.

In the proposed model parameterization, the training parameters are either a subset of the Bezier CPs or a single parameter defining the CP coordinates simultaneously. Specifically, the parameter L is defined that controls the extension of the curve. The x coordinates of the CPs are equally spaced between [0,L]. Moreover, the training parameters $y_0$ and $y_L$ are defined which are set equal to the y CP coordinates of the first and last 3 points, respectively. This allows for smooth continuous first and second derivatives at the edges of the curve. Each of the y coordinates of the rest of the CPs are considered as independent training parameters. Therefore, the CPs are defined as

$$\begin{aligned} b_y^0 = b_y^1 = b_y^2 &:= y_0 \\ b_y^{N_b} = b_y^{N_b-1} = b^{N_b-2} &:= y_L \\ b_y^{m+2} &:= y_m \ \forall\ m = 1,\dots,N_b-5 \\ b_x^m &:= \frac{mL}{N_b} \ \forall\ m = 0,\dots,N_b \end{aligned} \tag{S3}$$

where $\mathbf{k}$ :=(L, $y_0$, $y_1$,..., $y_{N_b-5}, y_L$) are the respective optimization parameters. It is also worth mentioning that given equally spaced x CPs between 0 and L, x(t) essentially drops to

$$x(t) = Lt \tag{S4}$$

which is very convenient, since we can calculate analytically $t$ from the given feature or descriptor $x$, later used in the calculation of y(t), which in this context is a contribution to the total interaction energy.

Figure S2 (left pannel) demonstrates the construction of the Bezier curve with ($y_0$, $y_L$ , L)=(0,0,10). The curves begin at zero and end at L=10. Changing slightly the y positions of the internal CPs ($y_1$ to $y_{N_b-5}$) produces a slightly different curve. Figure S2 (middle panel) shows the effect of varying L, while the effect of varying $y_0$ and $y_L$ is shown in Figure S2 (right panel).

The code may treat any model parameter as model parameter to be optimized or fix it at a specific value. To give some guidance, one would prefer to keep fix y0=0 for LD embedding functions, since at zero density the contribution is zero. In another example, for pairwise interactions, one should allow y0 to vary to capture and correct the strong repulsive part and fix L in order to avoid artificial long range interactions. Hence, this parameterization framework is well suited for well-controlled potential forms given chemical or mathematical intuition.

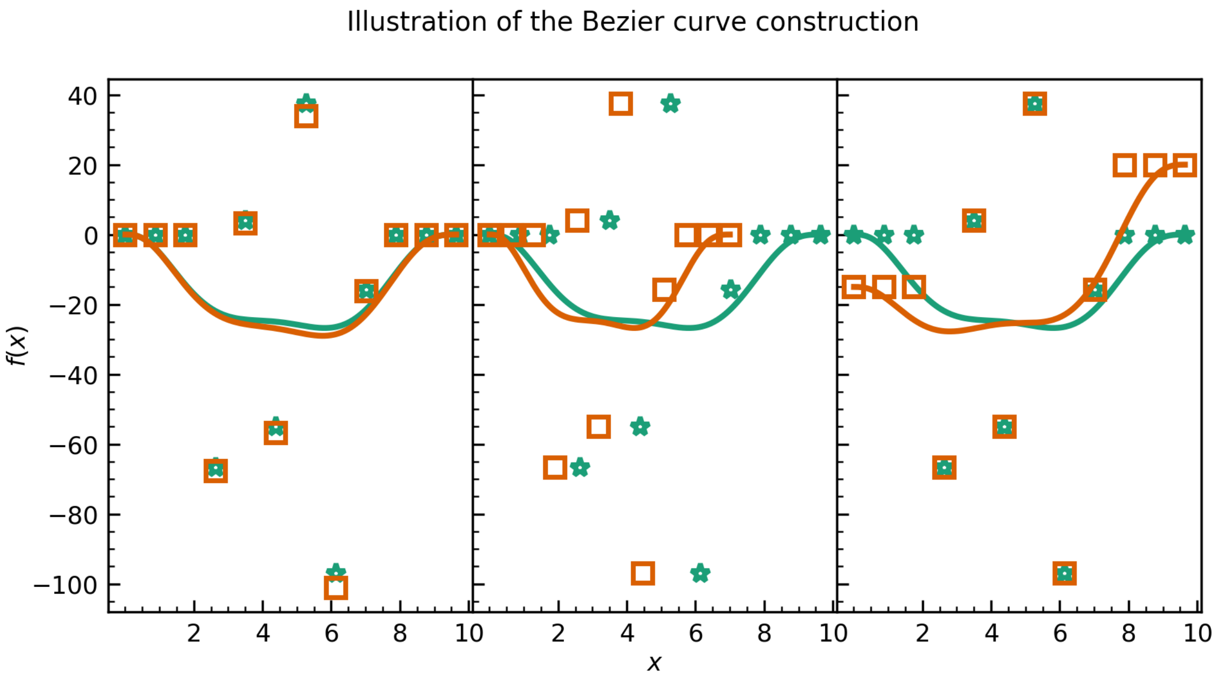


Figure S2: Illustration of the Bezier curve parameterization. Orange squares are the Bezier CPs that shape the orange curve, while the green stars are the CPs shaping the green curves. Left panel: the effect of varying the internal points y position. Middle panel: The effect of varying L and hence the x position of all the CPs. Right panel: The effect of varying the y0 and yL parameters and hence the three initial and three last Bezier CPs.

# S3 Outlier Score Computation

Here, we demonstrate the computation of the outlier score (OS) in Figure S3. In all cases, large OS values ($> 1$) for a specific descriptor denote structures that are of the distribution of the same descriptor in the existing dataset. The left panel shows a case of a narrow distribution where OS can take values much higher than one due to the small value of $\omega_{max} - \omega_{min}$ (see main text definitions). The middle and left panels show that even in multimodal distributions, high OS values are assigned for all low values of $h(x)$, even if $x'$ is between two modes. Outside of the distribution range $OS > 1$.

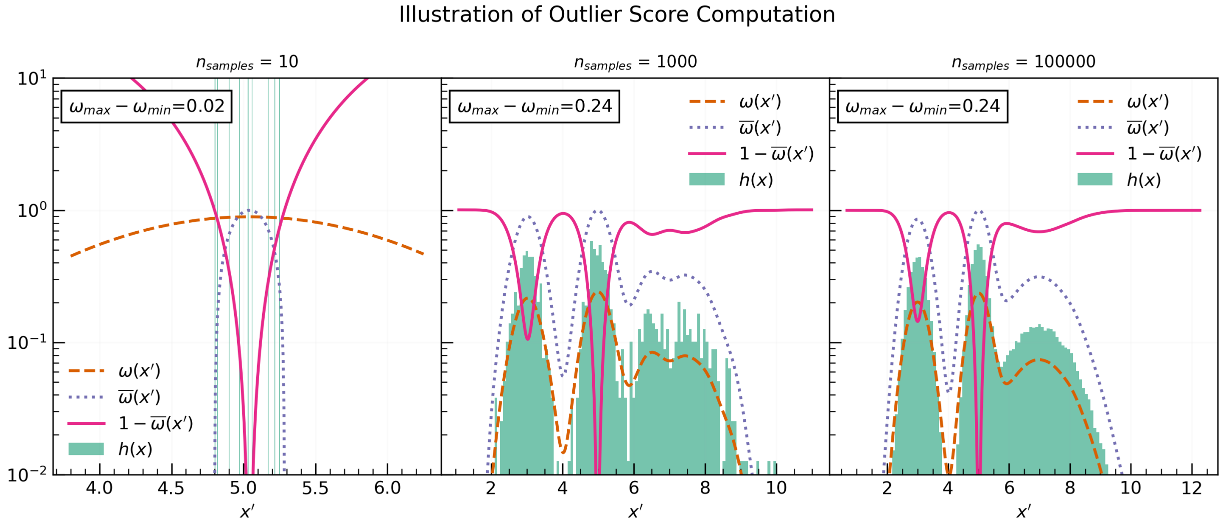


Figure S3: Illustration of the Outlier Score (OS) computation in increasing numbers of samples ($n_{samples}$) in example distributions $h(x)$. The left panel corresponds to a narrow distribution, possessing very low $\omega_{max} - \omega_{min}$ values. The middle and right panels correspond to distributions where $\omega_{max} - \omega_{min}$ is higher. Data outside of the range of the distribution are assigned $OS(x') := 1 - \overline{\omega}(x') > 1$, while rare data between the modes of the distribution get values close 1. Values at the peak of the distribution get $OS$ values close to zero.

# S4 Discussion on the Training Method

Figures S4(a-c) demonstrate the effect of minimizing the energy cost $C_E$ at descending values of $C_F$. The best trade-off solutions for the $2^{nd}$ and $3^{rd}$ AL iterations, are significantly better than the initial ones, while a lot of non-Pareto optimal solutions are found, indicating overfitting cases, due to the initial small training data sets. In fact, for the $3^{rd}$ AL iteration only one Pareto optimal

solution is found. For the 20$^{th}$ AL iteration, 4 Pareto optimal solutions are found from which the one with the best trade-off is selected. The lack of data in the early iterations makes the optimization landscape rougher, with multiple local solutions, however this algorithm is able to find better solutions by constraining $C_F$ at descending values. Given adequate data, we can calculate Pareto optimal solutions and select the one that preserves the best trade-off between $C_E$ and $C_F$.

Figure S5 illustrates the evolution of model parameters as a function of the active learning (AL) iteration. During the initial AL iterations, the parameters exhibit substantial variations between successive iterations. However, as the AL process progresses, these variations diminish, and the parameters either converge or undergo minor refinements. Additionally, the parameters are updated simultaneously and may occasionally undergo abrupt changes, shifting to significantly different solutions. A notable example occurs between the 11$^{th}$ and 12$^{th}$ AL iterations, where the parameters governing the Ag-gas interaction (specifically the Pairwise and Local Density terms) undergo substantial refinement. This behavior can be attributed to a significant enrichment of the dataset at the 12$^{th}$ AL iteration, following the algorithm's exploration of new regions of the potential energy surface (PES) in the preceding iteration. Indeed, at the 11$^{th}$ AL iteration, we observed elevated outlier scores for the selected batch and increased prediction errors associated with the $Ag_7/CO_2$ system.

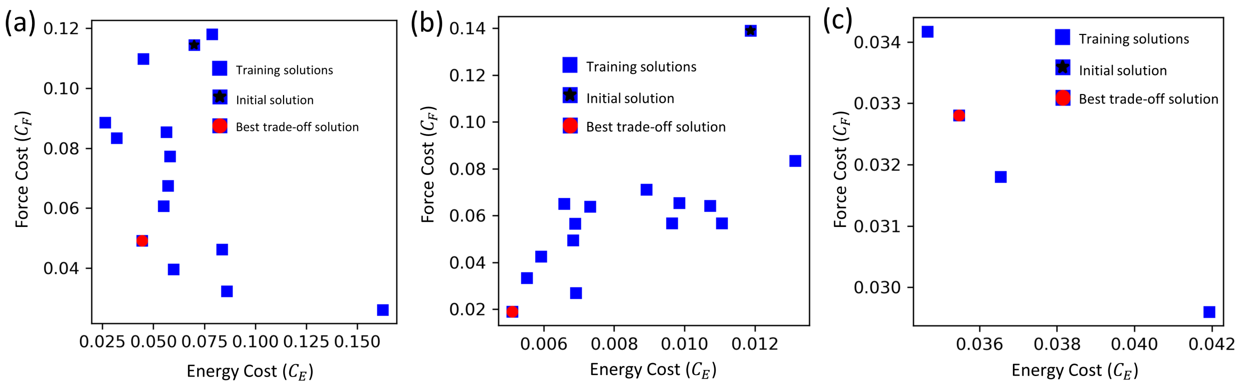


Figure S4: Training solutions by minimizing the energy cost $C_E$ at descending values of force cost $C_F$ at (a) the 2$^{nd}$, (b) 3$^{rd}$ and (c) 20$^{th}$ active learning iteration, for the $Ag_7CO_2$ hybrid and the isolated $Ag_7$ cluster. Since $C_E$ and $C_F$ are normalized they are dimensionless

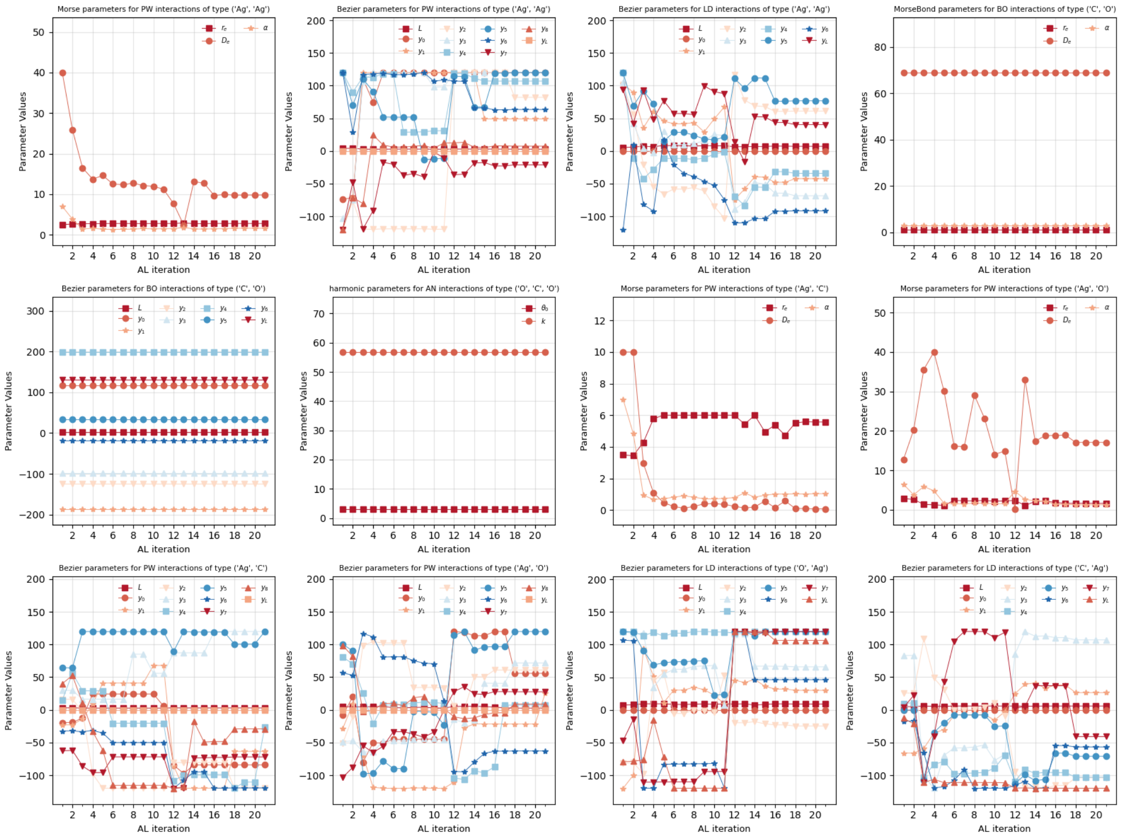


Figure S5: Parameters evolution and refinement as a function of AL iteration, for the $Ag_7$ and $Ag_7CO_2$ systems (case 1). First row panels depict $CO_2$ covalent, bond (BO) and bond angle (AN), potential parameters that were optimized independently and held fixed during this AL optimization. Second and third row panels depict non-bonded pairwise (PW) interaction parameters for Morse and Bezier functions, respectively. The fourth row panels depict the Bezier parameters for the local density (LD) interactions.

# S5 General Applicability

Figures S6-S8 present the predicted MAE and batch OS statistics as functions of AL for Cases 2-4. The OS shows similar convergence and correlation with MAE. In Case 2, the model achieves high accuracy (<10 meV/atom) after 21 iterations. Case 3 captures complex chemisorption with an acceptable error (3̃0 meV/atom), balancing simplicity and efficiency. Case 4 demonstrates a unified FF model for cluster sizes 9-16, with a trade-off in accuracy (MAE: 10-35 meV/atom).

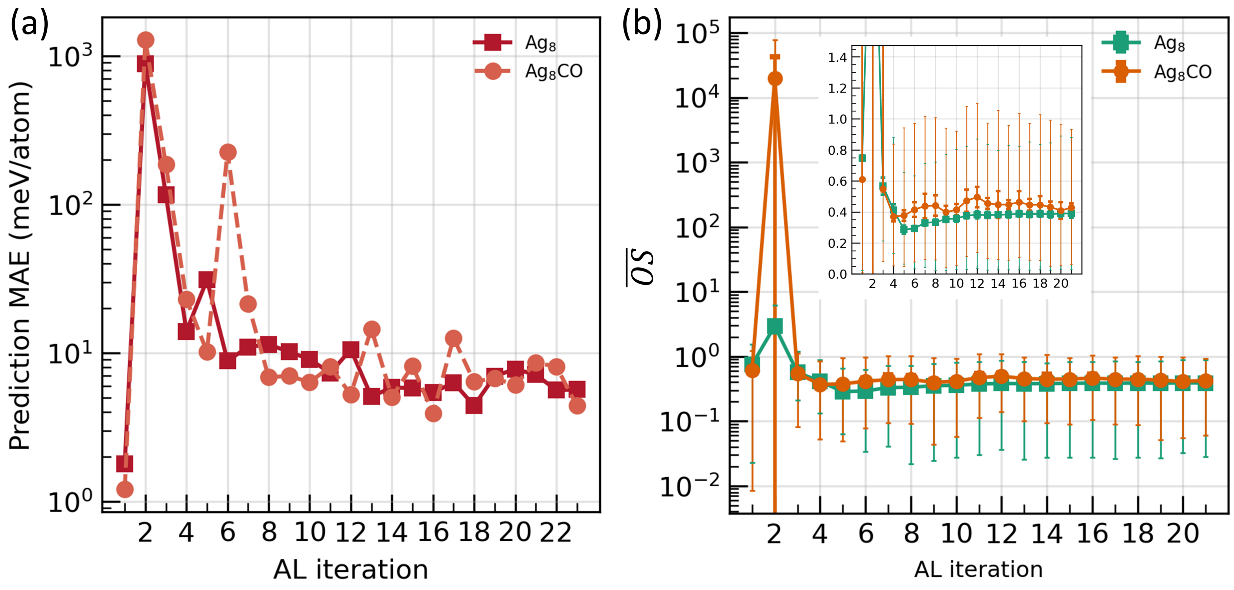


Figure S6: (a) Prediction MAE and (b) calculated outlier score statistics (mean, standard deviation, maximum, minimum) as a function of AL iteration, per system, for systems $Ag_8$ and $Ag_8CO$. Thick caps denote standard deviation, while thin caps the maximum and minimum values.

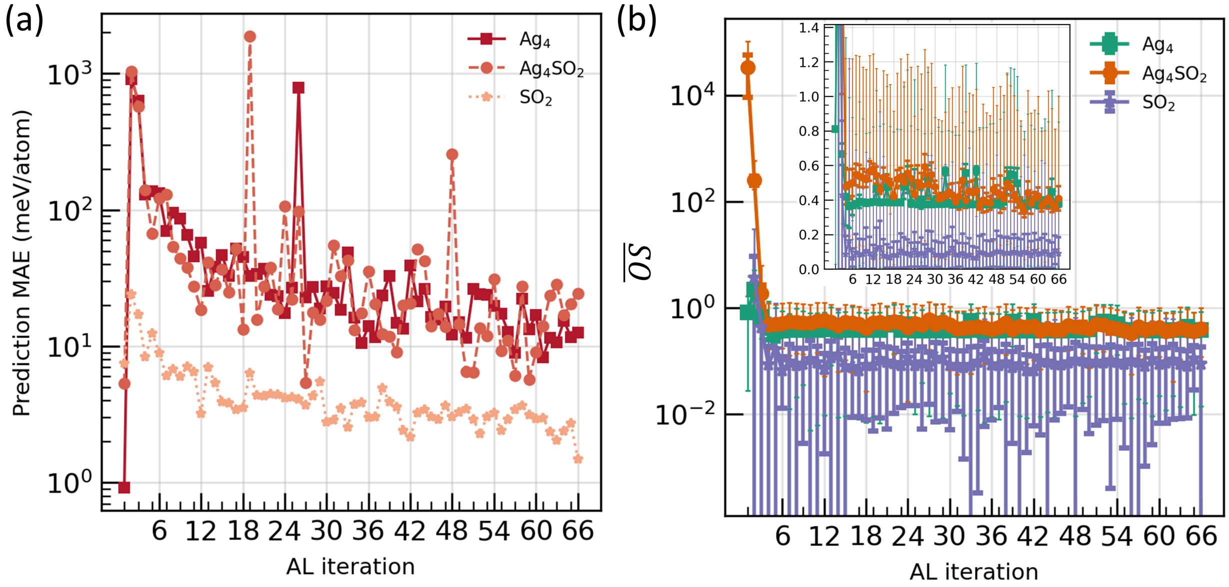


Figure S7: (a) Prediction MAE and (b) calculated outlier score statistics (mean, standard deviation, maximum, minimum) as a function of AL iteration, per system, for systems $Ag_4SO_2$ and isolated $Ag_4$ and $SO_2$. Thick caps denote standard deviation, while thin caps the maximum and minimum values.

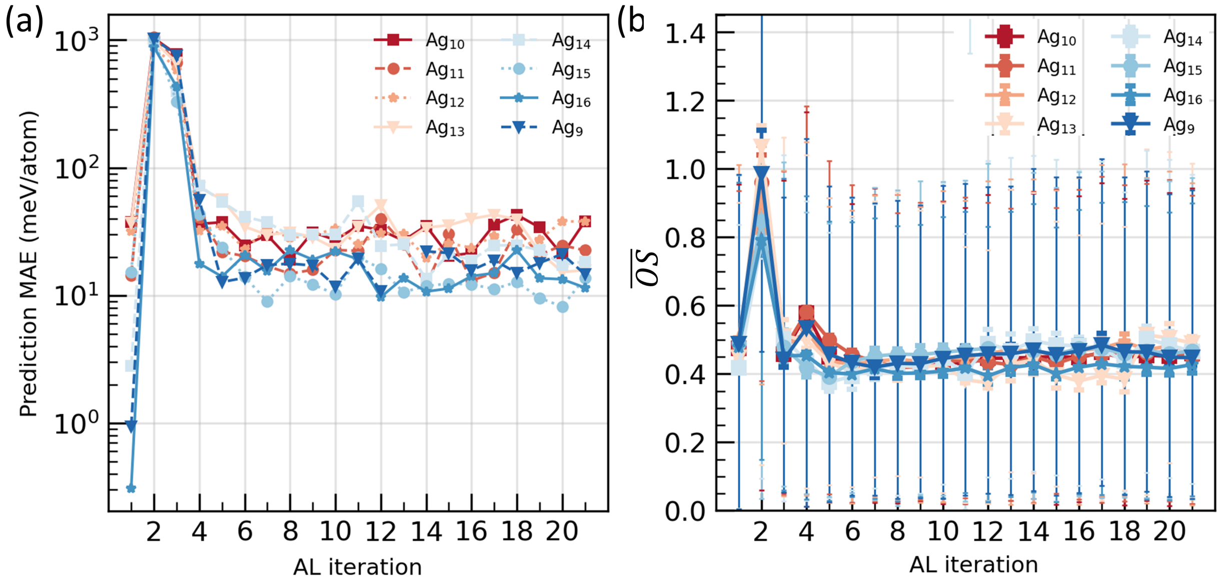


Figure S8: (a) Prediction MAE and (b) calculated outlier score statistics (mean, standard deviation, maximum, minimum) as a function of AL iteration, per system, for systems $Ag_9$, $Ag_{10}$, $Ag_{11}$, $Ag_{12}$, $Ag_{13}$, $Ag_{14}$, $Ag_{15}$ and $Ag_{16}$ . Thick caps denote standard deviation, while thin caps the maximum and minimum values.

# S6 Optimized Force Field Parameter Tables

The final optimized parameters for Cases 1-5 (see main text) are detailed in Tables S1-S5, respectively. For example, parameters in Table S1 are the last AL iteration parameters depicted in Figure S2. Types are denoted as pairs in a parenthesis (*e.g.,* ('O', 'Ag')) denotes interactions between O and Ag atoms. There are 4 interaction "categories" in this model, pairwise (PW), denoting pairwise non-bonded interactions, LD, capturing the many body effects in an efficient manner, covalent bond (BO), for any predefined covalent bonds, and bond angle (AN) for any predefined covalent angle in the system. In case of local density (LD) interactions the atom on the left of the parenthesis denotes the central atom.

Table S1: Potential information and parameters for case 1 ($Ag_7$ and $Ag_7CO_2$ systems). Abbreviations: Pairwise (PW), Local Density (LD), covalent bond (BO), bond angle (AN). Central atoms in LD are written to the left of the parenthesis.

| Term | Category | Types | Function | Function Parameters |
|---|---|---|---|---|
| 1 | PW | ('Ag', 'Ag') | Morse | $r_e$ = 2.787, $D_e$ = 9.809, $\alpha$ = 1.603 |
| 2 | PW | ('Ag', 'Ag') | Bezier | $L$ = 2.762, $y_0$ = 119.792, $y_1$ = 49.054, $y_2$ = 82.082, $y_3$ = 119.769, $y_4$ = 106.904, $y_5$ = 119.988, $y_6$ = 63.474, $y_7$ = -21.259, $y_8$ = 7.315, $y_L$ = 0.000 |
| 3 | LD | ('Ag', 'Ag') | Bezier | $L$ = 7.088, $y_0$ = 0.000, $y_1$ = -42.444, $y_2$ = 61.078, $y_3$ = -68.610, $y_4$ = -34.323, $y_5$ = 76.841, $y_6$ = -91.611, $y_L$ = 40.258 |
| 4 | BO | ('C', 'O') | Morse (Bond) | $r_e$ = 1.263, $D_e$ = 68.982, $\alpha$ = 2.828 |
| 5 | BO | ('C', 'O') | Bezier | $L$ = 2.405, $y_0$ = 117.003, $y_1$ = -185.990, $y_2$ = -124.585, $y_3$ = -98.413, $y_4$ = 198.696, $y_5$ = 33.893, $y_6$ = -18.108, $y_L$ = 131.257 |
| 6 | AN | ('O', 'C', 'O') | harmonic | $\theta_0$ = 3.142, $k$ = 56.809 |
| 7 | PW | ('Ag', 'C') | Morse | $r_e$ = 5.570, $D_e$ = 0.073, $\alpha$ = 1.034 |
| 8 | PW | ('Ag', 'O') | Morse | $r_e$ = 1.638, $D_e$ = 17.090, $\alpha$ = 1.279 |
| 9 | PW | ('Ag', 'C') | Bezier | $L$ = 2.966, $y_0$ = -84.085, $y_1$ = -63.378, $y_2$ = -119.846, $y_3$ = 119.850, $y_4$ = -25.856, $y_5$ = 119.880, $y_6$ = -119.308, $y_7$ = -71.910, $y_8$ = -29.054, $y_L$ = 0.000 |
| 10 | PW | ('Ag', 'O') | Bezier | $L$ = 2.705, $y_0$ = 55.980, $y_1$ = 25.227, $y_2$ = 60.954, $y_3$ = 71.753, $y_4$ = 7.288, $y_5$ = 119.814, $y_6$ = -62.791, $y_7$ = 27.728, $y_8$ = 8.864, $y_L$ = 0.000 |
| 11 | LD | ('O', 'Ag') | Bezier | $L$ = 9.014, $y_0$ = 0.000, $y_1$ = 29.991, $y_2$ = -24.674, $y_3$ = 65.888, $y_4$ = 119.559, $y_5$ = 119.860, $y_6$ = 46.251, $y_7$ = 119.794, $y_L$ = 106.331 |
| 12 | LD | ('C', 'Ag') | Bezier | $L$ = 5.920, $y_0$ = 0.000, $y_1$ = 26.392, $y_2$ = -103.896, $y_3$ = 107.077, $y_4$ = -102.999, $y_5$ = -70.961, $y_6$ = -56.722, $y_7$ = -40.770, $y_L$ = -119.901 |

Table S2: Potential information and parameters for case 2 ($Ag_8$ and $Ag_8CO$ systems). Abbreviations: Pairwise (PW), Local Density (LD), covalent bond (BO). Central atoms in LD are written to the left of the parenthesis.

| Term | Category | Types | Function | Function Parameters |
|---|---|---|---|---|
| 1 | BO | ('C', 'O') | Morse (Bond) | $r_e$ = 1.211, $D_e$ = 191.920, $\alpha$ = 2.123 |
| 2 | BO | ('C', 'O') | Bezier | $L$ = 2.112, $y_0$ = 191.968, $y_1$ = -139.935, $y_2$ = -162.652, $y_3$ = -100.452, $y_4$ = 168.532, $y_5$ = 76.527, $y_L$ = 9.840 |
| 3 | PW | ('Ag', 'C') | Morse | $r_e$ = 2.854, $D_e$ = 0.016, $\alpha$ = 3.159 |
| 4 | PW | ('Ag', 'O') | Morse | $r_e$ = 2.114, $D_e$ = 8.764, $\alpha$ = 5.291 |
| 5 | PW | ('Ag', 'C') | Bezier | $L$ = 4.070, $y_0$ = 64.412, $y_1$ = 100.566, $y_2$ = -81.916, $y_3$ = -76.554, $y_4$ = 90.298, $y_5$ = -44.661, $y_6$ = 1.974, $y_L$ = 0.000 |
| 6 | PW | ('Ag', 'O') | Bezier | $L$ = 3.390, $y_0$ = 0.923, $y_1$ = 119.113, $y_2$ = 102.282, $y_3$ = 114.400, $y_4$ = -2.563, $y_5$ = 19.973, $y_6$ = 14.220, $y_L$ = 0.000 |
| 7 | PW | ('Ag', 'Ag') | Morse | $r_e$ = 2.942, $D_e$ = 0.386, $\alpha$ = 3.091 |
| 8 | PW | ('Ag', 'Ag') | Bezier | $L$ = 5.000, $y_0$ = 1.520, $y_1$ = 36.354, $y_2$ = 45.687, $y_3$ = 108.069, $y_4$ = -100.660, $y_5$ = 2.087, $y_6$ = 60.402, $y_7$ = -48.816, $y_8$ = 12.643, $y_L$ = -12.248 |
| 9 | LD | ('Ag', 'Ag') | Bezier | $L$ = 7.641, $y_0$ = 0.000, $y_1$ = 96.003, $y_2$ = -60.696, $y_3$ = -63.677, $y_4$ = 94.100, $y_5$ = -96.541, $y_6$ = -39.023, $y_L$ = -54.028 |
| 10 | LD | ('O', 'Ag') | Bezier | $L$ = 7.445, $y_0$ = 0.000, $y_1$ = -37.353, $y_2$ = 64.649, $y_3$ = -48.294, $y_4$ = 6.111, $y_5$ = 28.591, $y_6$ = 119.269, $y_L$ = 15.205 |
| 11 | LD | ('C', 'Ag') | Bezier | $L$ = 7.903, $y_0$ = 0.000, $y_1$ = 15.421, $y_2$ = -29.992, $y_3$ = 10.992, $y_4$ = 119.973, $y_5$ = 15.143, $y_6$ = 119.147, $y_L$ = 57.988 |

Table S3: Potential information and parameters for case 3 ($Ag_4SO_2$ and isolated $Ag_4$ and $SO_2$ systems). Abbreviations: Pairwise (PW), Local Density (LD), covalent bond (BO), bond angle (AN). Central atoms in LD are written to the left of the parenthesis.

| Term | Category | Types | Function | Function Parameters |
|---|---|---|---|---|
| 1 | BO | ('O', 'S') | Morse (Bond) | $r_e$ = 1.815, $D_e$ = 6.189, $\alpha$ = 2.850 |
| 2 | BO | ('O', 'S') | Bezier | $L$ = 2.618, $y_0$ = 150.501, $y_1$ = -182.991, $y_2$ = -196.790, $y_3$ = 29.929, $y_4$ = -199.515, $y_5$ = 169.016, $y_6$ = 100.316, $y_L$ = 0.000 |
| 3 | AN | ('O', 'S', 'O') | harmonic | $\theta_0$ = 2.067, $k$ = 98.532 |
| 4 | PW | ('Ag', 'S') | Morse | $r_e$ = 5.600, $D_e$ = 2.831, $\alpha$ = 0.595 |
| 5 | PW | ('Ag', 'O') | Morse | $r_e$ = 2.011, $D_e$ = 26.209, $\alpha$ = 1.929 |
| 6 | PW | ('Ag', 'O') | Bezier | $L$ = 3.299, $y_0$ = 192.012, $y_1$ = 178.789, $y_2$ = 72.579, $y_3$ = -194.039, $y_4$ = -107.166, $y_5$ = 184.875, $y_6$ = 14.502, $y_7$ = -75.904, $y_8$ = 34.858, $y_L$ = 0.000 |
| 7 | PW | ('Ag', 'S') | Bezier | $L$ = 2.939, $y_0$ = -199.755, $y_1$ = 175.148, $y_2$ = -29.421, $y_3$ = 162.145, $y_4$ = -196.591, $y_5$ = -62.667, $y_6$ = -188.546, $y_7$ = -112.249, $y_L$ = 0.000 |
| 8 | LD | ('O', 'Ag') | Bezier | $L$ = 8.816, $y_0$ = 0.000, $y_1$ = 24.959, $y_2$ = 103.479, $y_3$ = -85.263, $y_4$ = -27.171, $y_5$ = 48.079, $y_6$ = -118.830, $y_L$ = 119.538 |
| 9 | LD | ('S', 'Ag') | Bezier | $L$ = 7.206, $y_0$ = 0.000, $y_1$ = 35.164, $y_2$ = -20.538, $y_3$ = 4.145, $y_4$ = -42.490, $y_5$ = 92.171, $y_6$ = -82.004, $y_L$ = 92.820 |
| 10 | PW | ('Ag', 'Ag') | Morse | $r_e$ = 2.709, $D_e$ = 19.462, $\alpha$ = 2.012 |
| 11 | PW | ('Ag', 'Ag') | Bezier | $L$ = 2.924, $y_0$ = -117.927, $y_1$ = 113.711, $y_2$ = 3.840, $y_3$ = -77.502, $y_4$ = -85.465, $y_5$ = 89.793, $y_6$ = 22.088, $y_7$ = -93.077, $y_8$ = 11.657, $y_L$ = 0.000 |
| 12 | LD | ('Ag', 'Ag') | Bezier | $L$ = 5.919, $y_0$ = 0.000, $y_1$ = 3.320, $y_2$ = -89.303, $y_3$ = 60.109, $y_4$ = 81.084, $y_5$ = 12.468, $y_6$ = -46.232, $y_L$ = -58.086 |

Table S4: Potential information and parameters for case 4 (systems $Ag_9$, $Ag_{10}$, $Ag_{11}$, $Ag_{12}$, $Ag_{13}$, $Ag_{14}$, $Ag_{15}$ and $Ag_{16}$ ). Abbreviations: Pairwise (PW), Local Density (LD).

| Term | Category | Types | Function | Function Parameters |
|---|---|---|---|---|
| 1 | PW | ('Ag', 'Ag') | Morse | $r_e$ = 3.027, $D_e$ = 12.555, $\alpha$ = 1.089 |
| 2 | PW | ('Ag', 'Ag') | Bezier | $L$ = 3.373, $y_0$ = -115.308, $y_1$ = 97.080, $y_2$ = 111.050, $y_3$ = -27.460, $y_4$ = 19.691, $y_5$ = 85.178, $y_6$ = 22.915, $y_7$ = -18.401, $y_8$ = 26.214, $y_9$ = -74.904, $y_{10}$ = 35.420, $y_{11}$ = -18.546, $y_L$ = 0.000 |
| 3 | LD | ('Ag', 'Ag') | Bezier | $L$ = 16.105, $y_0$ = 0.000, $y_1$ = 0.853, $y_2$ = -42.515, $y_3$ = 90.266, $y_4$ = 10.468, $y_5$ = -38.423, $y_6$ = 85.582, $y_7$ = -1.341, $y_8$ = 111.818, $y_9$ = 112.682, $y_{10}$ = 118.493, $y_{11}$ = 85.797, $y_L$ = 213.864 |

Table S5: Potential information and parameters refining the FF of case 4, for systems $Ag_{17}$ and $Ag_{18}$, in a "transfer learning" procedure. The 4$^{th}$ potential term is added while the rest are kept fixed as in Table S4 . Abbreviations: Pairwise (PW), Local Density (LD)

| Term | Category | Types | Function | Function Parameters |
|---|---|---|---|---|
| 1 | PW | ('Ag', 'Ag') | Morse | $r_e$ = 3.027, $D_e$ = 12.555, $\alpha$ = 1.089 |
| 2 | PW | ('Ag', 'Ag') | Bezier | $L$ = 3.373, $y_0$ = -115.308, $y_1$ = 97.080, $y_2$ = 111.050, $y_3$ = -27.460, $y_4$ = 19.691, $y_5$ = 85.178, $y_6$ = 22.915, $y_7$ = -18.401, $y_8$ = 26.214, $y_9$ = -74.904, $y_{10}$ = 35.420, $y_{11}$ = -18.546, $y_L$ = 0.000 |
| 3 | LD | ('Ag', 'Ag') | Bezier | $L$ = 16.105, $y_0$ = 0.000, $y_1$ = 0.853, $y_2$ = -42.515, $y_3$ = 90.266, $y_4$ = 10.468, $y_5$ = -38.423, $y_6$ = 85.582, $y_7$ = -1.341, $y_8$ = 111.818, $y_9$ = 112.682, $y_{10}$ = 118.493, $y_{11}$ = 85.797, $y_L$ = 213.864 |
| 4 | LD | ('Ag', 'Ag') | Bezier | $L$ = 6.000, $y_0$ = 0.000, $y_1$ = 46.777, $y_2$ = -58.717, $y_3$ = 69.431, $y_4$ = -54.681, $y_5$ = 40.597, $y_6$ = -16.821, $y_7$ = 3.508, $y_L$ = -0.849 |

# S7 Energy, Energy Fitting Error & Descriptor Distributions

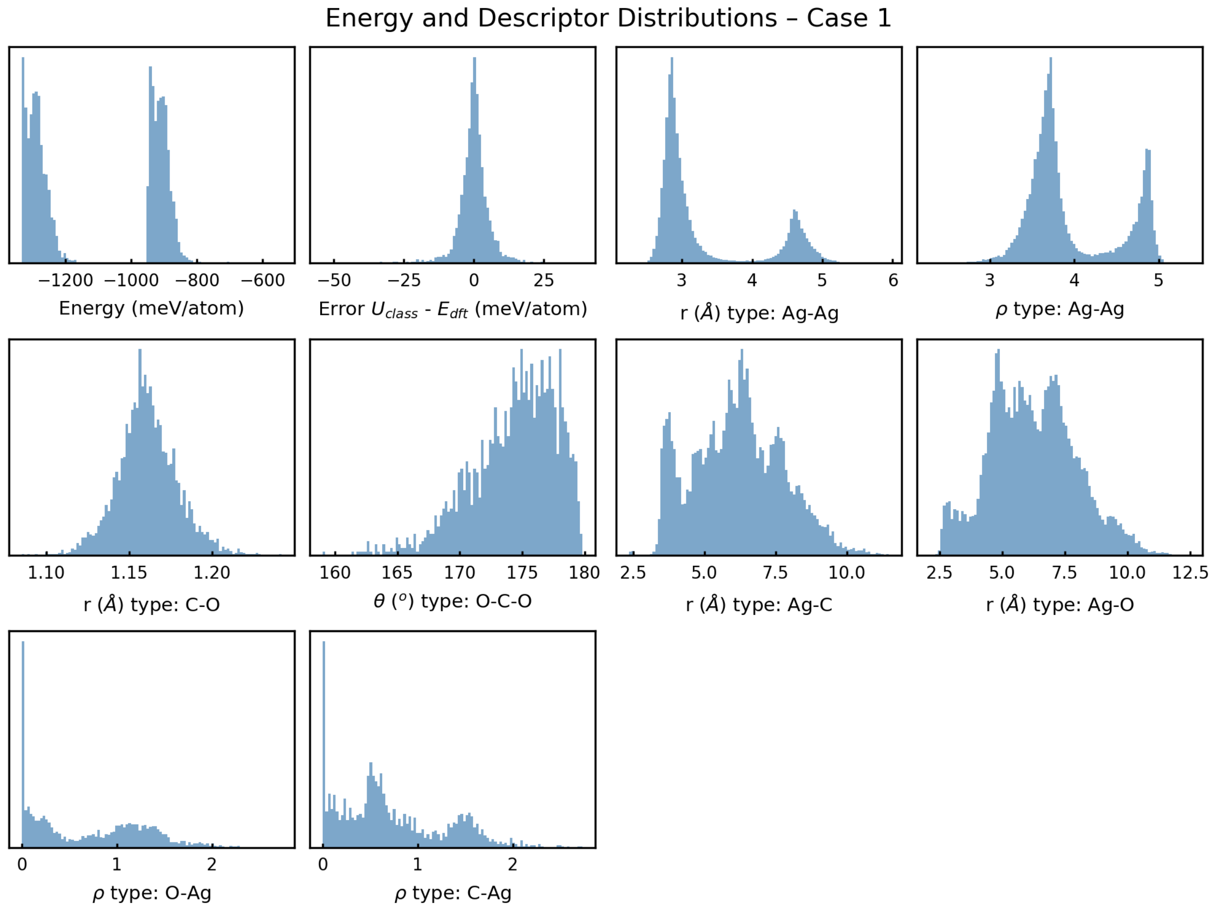


Figure S9: Energy, energy fitting error and descriptor distributions for case 1 (systems $Ag_7$, $Ag_7CO_2$)

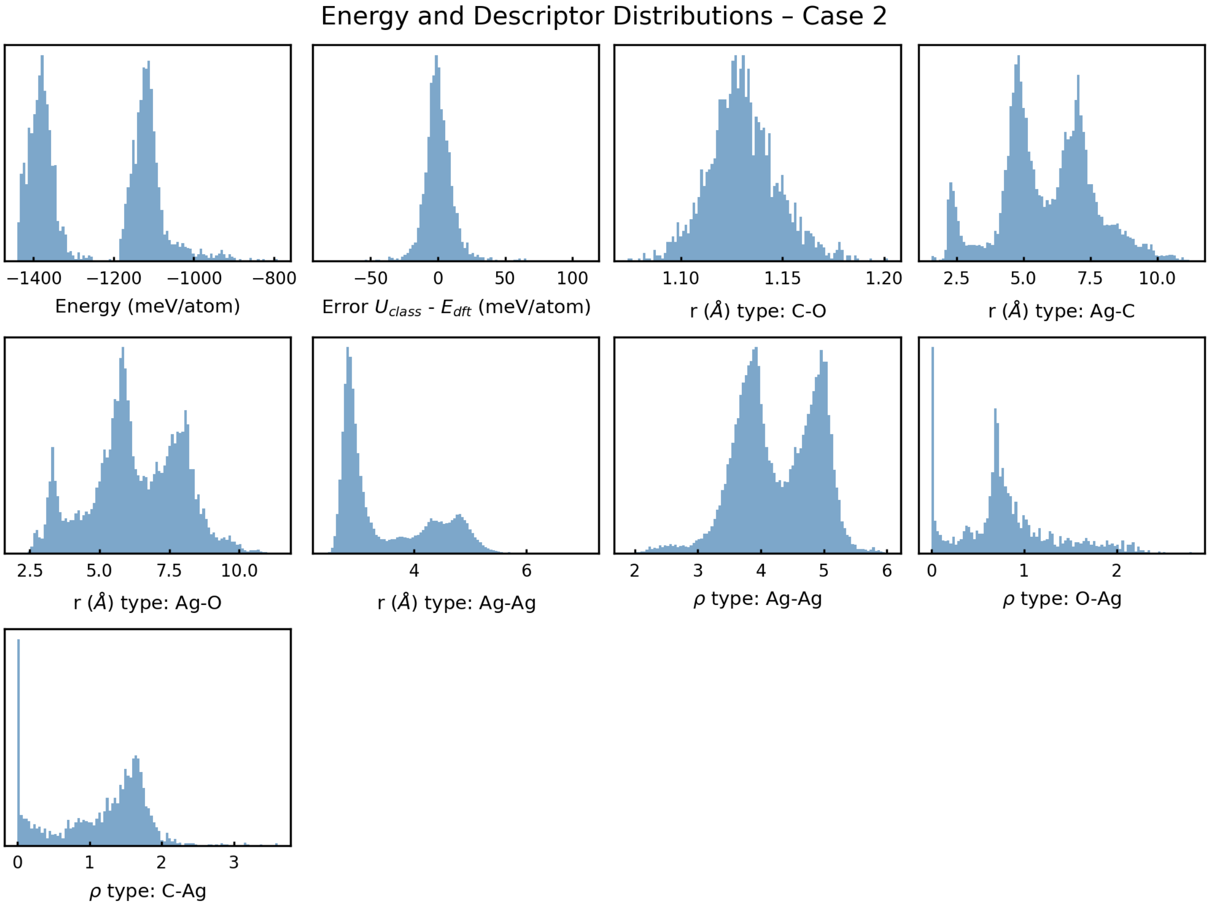


Figure S10: Energy, energy fitting error and descriptor distributions for case 2 (systems $Ag_8$, $Ag_8CO$)

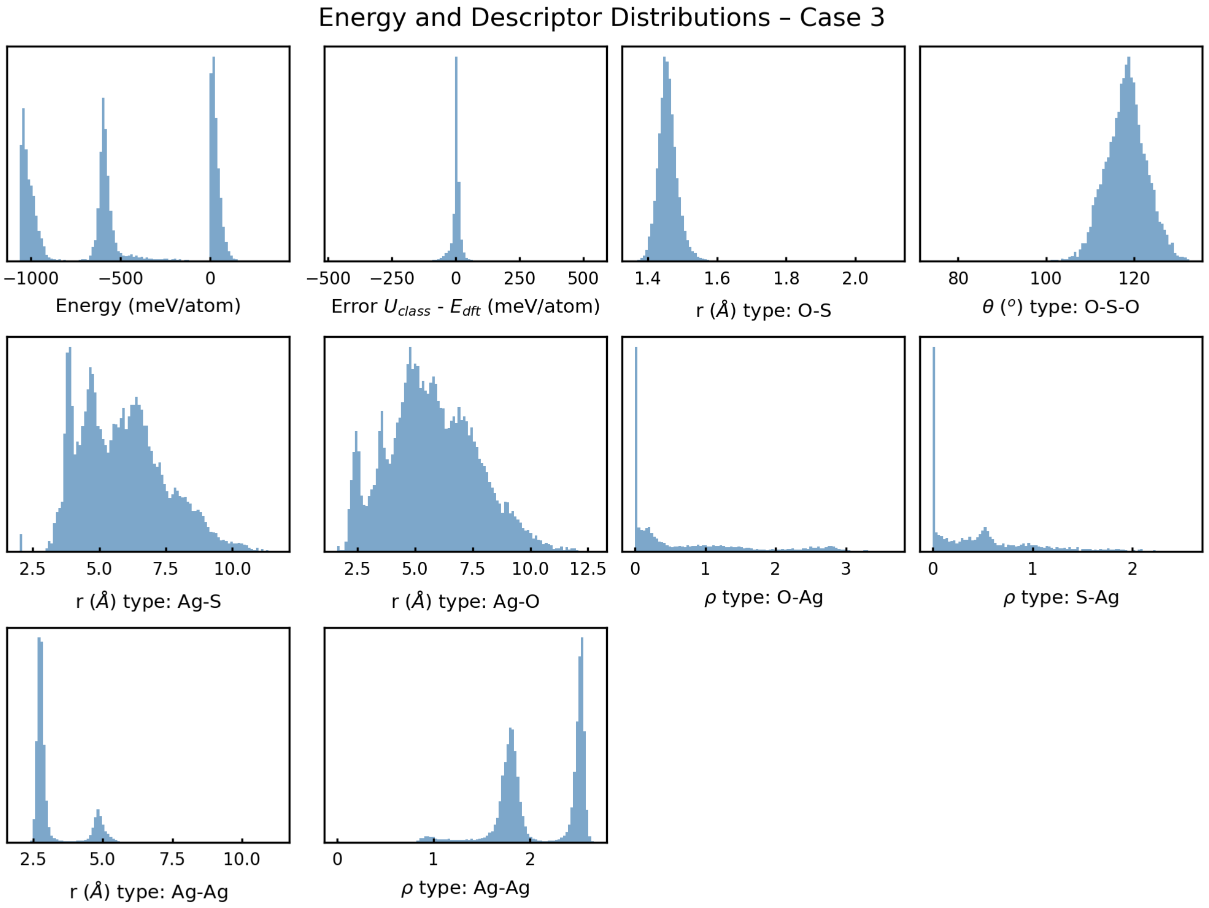


Figure S11: Energy, energy fitting error and descriptor distributions for case 3 (systems $Ag_4$, $Ag_4SO_2$)

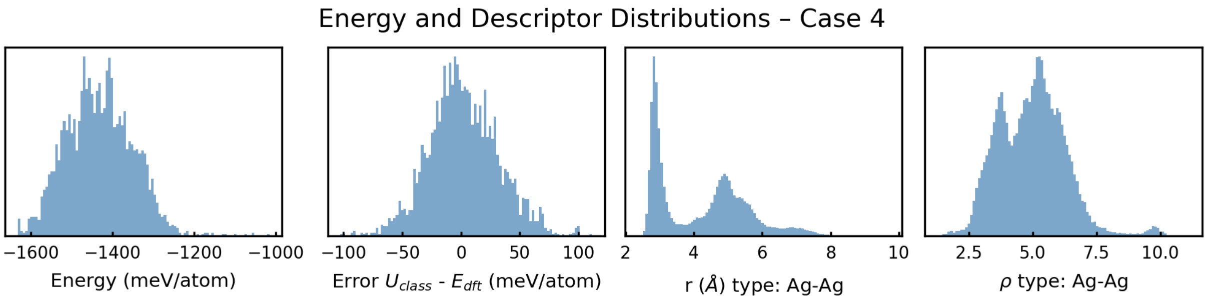


Figure S12: Energy, energy fitting error and descriptor distributions for case 4 (systems $Ag_9$-$Ag_{16}$)

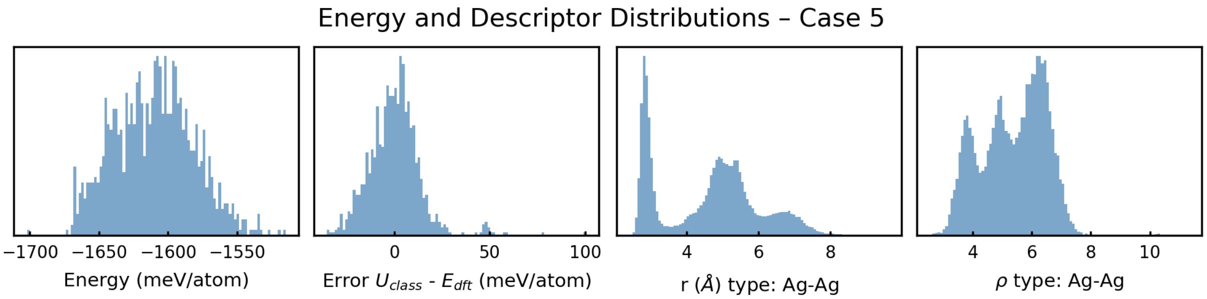


Figure S13: Energy, energy fitting error and descriptor distributions for case 5 (systems $Ag_{17}$, $Ag_{18}$)

# S8 Optimized Potential Plot for Case 5

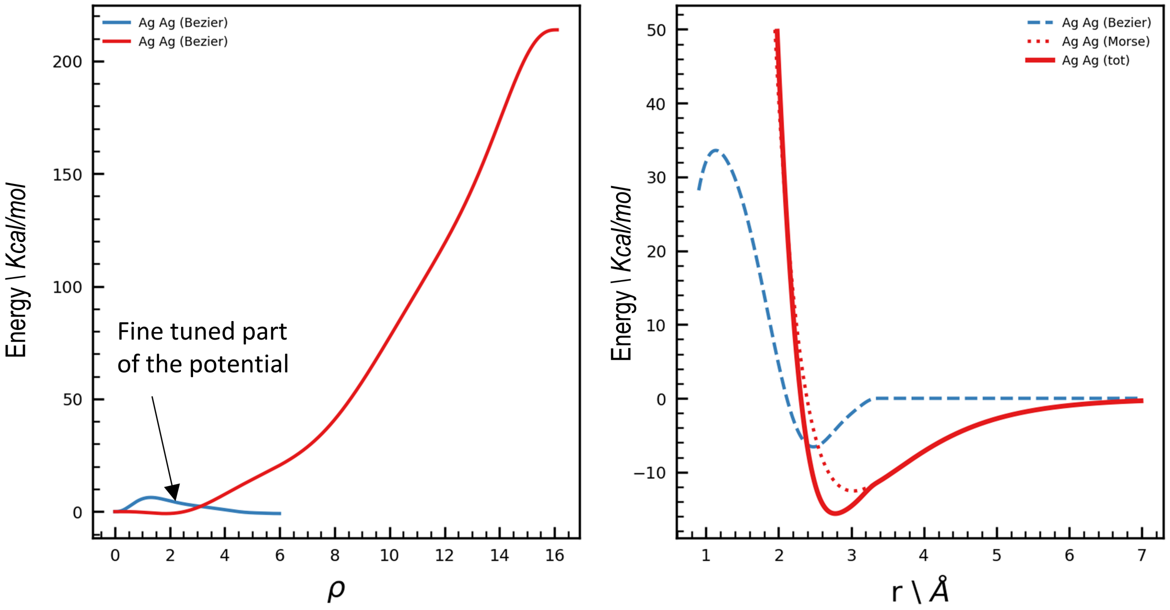


Figure S14: Potential profile for Case 5 ($Ag_{17}$, $Ag_{18}$ systems). The right panel shows the pairwise distance-based contributions to the potential, while the left panel depicts the embedding density-dependent components. The shorter curve represents the fine-tuned segment of the embedding potential. The remaining curves were obtained during the active learning procedure applied in Case 4 ($Ag_9$–$Ag_{16}$ systems).
